# Psychological Support Strategies for Adults With Type 2 Diabetes in a Very Low–Carbohydrate Web-Based Program: Randomized Controlled Trial

**DOI:** 10.2196/44295

**Published:** 2023-05-11

**Authors:** Laura R Saslow, Amanda L Missel, Alison O’Brien, Sarah Kim, Frederick M Hecht, Judith T Moskowitz, Hovig Bayandorian, Martha Pietrucha, Kate Raymond, Blair Richards, Bradley Liestenfeltz, Ashley E Mason, Jennifer Daubenmier, James E Aikens

**Affiliations:** 1 Department of Health Behavior and Biological Sciences School of Nursing University of Michigan Ann Arbor, MI United States; 2 Department of Learning Health Sciences University of Michigan Medical School Ann Arbor, MI United States; 3 Institute for Healthcare Policy and Innovation University of Michigan Ann Arbor, MI United States; 4 Division of Endocrinology, Diabetes and Metabolism Department of Medicine San Francisco General Hospital San Francisco, CA United States; 5 Osher Center for Integrative Health University of California San Francisco San Francisco, CA United States; 6 Feinberg School of Medicine Northwestern University Chicago, IL United States; 7 Michigan Institute for Clinical and Health Research University of Michigan Ann Arbor, MI United States; 8 Department of Psychiatry University of California San Francisco San Francisco, CA United States; 9 Institute of Holistic Health Studies San Francisco State University San Francisco, CA United States; 10 Department of Family Medicine University of Michigan Medical School Ann Arbor, MI United States

**Keywords:** eHealth, type 2 diabetes, T2D, very low–carbohydrate diet, weight loss, glycemic control, text messages, self-monitoring

## Abstract

**Background:**

A very low–carbohydrate (VLC) nutritional strategy may improve glycemic control and weight loss in adults with type 2 diabetes (T2D). However, the supplementary behavioral strategies that might be able to improve outcomes using this nutritional strategy are uncertain.

**Objective:**

This study aims to compare the impact of adding 3 different supplementary behavioral strategies to a web-based VLC diet intervention. To our knowledge, this is the first trial to randomize participants to different frequencies of dietary self-monitoring.

**Methods:**

The study included 112 overweight adults with T2D (hemoglobin A_1c_ ≥6.5%) taking no antiglycemic medications or only metformin. They received a remotely delivered 12-month VLC diet intervention. Participants were randomly assigned through a full factorial 2×2×2 design to supplementary strategies: either daily or monthly dietary self-monitoring, either mindful eating training or not, and either positive affect skills training or not. Our research goal was to determine whether 3 different supplemental strategies had at least a medium effect size (Cohen *d*=0.5).

**Results:**

Overall, the VLC intervention led to statistically significant improvements in glycemic control (−0.70%, 95% CI −1.04% to −0.35%; *P*<.001), weight loss (−6.82%, 95% CI −8.57% to −5.08%; *P*<.001), and depressive symptom severity (Cohen *d* −0.67, 95% CI −0.92 to −0.41; *P*<.001). Furthermore, 30% (25/83) of the participants taking metformin at baseline reduced or discontinued their metformin. Only 1 Cohen *d* point estimate reached 0.5; daily (vs monthly) dietary self-monitoring had a worse impact on depressive symptoms severity (Cohen *d*=0.47, 95% CI −0.02 to 0.95; *P*=.06). None of the strategies had a statistically significant effect on outcomes. For changes in our primary outcome, hemoglobin A_1c_, the daily (vs monthly) dietary self-monitoring impact was 0.42% (95% CI −0.28% to 1.12%); for mindful eating, it was −0.47% (95% CI −1.15% to 0.22%); and for positive affect, it was 0.12% (95% CI −0.57% to 0.82%). Other results for daily (vs monthly) dietary self-monitoring were mixed, suggesting an increase in weight (0.98%) and depressive symptoms (Cohen *d*=0.47), less intervention satisfaction (Cohen *d*=−0.20), more sessions viewed (3.02), and greater dietary adherence (Cohen *d*=0.24). For mindful eating, the results suggested a benefit for dietary adherence (Cohen *d*=0.24) and intervention satisfaction (Cohen *d*=0.30). For positive affect, the results suggested a benefit for depressive symptoms (Cohen *d*=−0.32), the number of sessions viewed (3.68), dietary adherence (Cohen *d*=0.16), and intervention satisfaction (Cohen *d*=0.25).

**Conclusions:**

Overall, our results support the use of a VLC diet intervention in adults with T2D. The addition of monthly (not daily) dietary self-monitoring, mindful eating, and positive affect skills training did not show a definitive benefit, but it is worth further testing.

**Trial Registration:**

ClinicalTrials.gov NCT03037528; https://clinicaltrials.gov/ct2/show/NCT03037528

## Introduction

Type 2 diabetes (T2D) is one of the most prevalent contemporary public health problems in the United States. If the current trajectory of prevalence continues, 1 in 3 US adults will have T2D by 2050 [1]. Nutritional management is one of the cornerstones of T2D treatment, and several dietary approaches are recommended for T2D, including a very low–carbohydrate (VLC) diet [2]. For example, a report by the American Diabetes Association’s Nutrition Review Committee noted the benefits of a VLC diet and updated the policy guidelines to recommend that for people with T2D “...not meeting glycemic targets or where reducing antiglycemic medications is a priority, reducing overall carbohydrate intake with a low- or VLC eating plan is a viable approach” [3]. Physiologically, carbohydrate intake increases blood glucose levels, which, in turn, increase insulin secretion from the pancreas. Insulin then inhibits lipolysis and the subsequent release of fatty acids from cells [4]. Numerous studies indicate that VLC diets can be effective at improving glycemic control, reducing the need for glucose-lowering medications, and increasing weight loss in adults with T2D [5-8].

However, long-term adherence to any behavioral intervention can be challenging, and the behavioral strategies that may help improve the outcomes of VLC interventions are unclear. In this trial, we screened 3 potentially effective supplemental behavioral strategies using a full factorial design. This was informed by the Multiphase Optimization Strategy framework [9]. This approach suggests that before conducting large clinical trials of multicomponent interventions, particular aspects of the intervention should be tested, especially those that might be costly, burdensome, or simply have not been previously tested enough to be clearly appropriate for a particular intervention. Such an approach is becoming more common in behavioral intervention development, for example, in the areas of weight loss [10], physical activity promotion [11], and our previous pilot study of the VLC diet for adults with T2D [12].

In this trial, we examined 3 supplemental strategies that were low-cost and varied in their degree of burden and level of previous testing. The first strategy we tested was dietary self-monitoring, wherein we varied whether we encouraged participants to practice dietary self-monitoring daily versus monthly (with monthly being defined as monitoring one’s diet in bursts of 3 days every 4 weeks). Weight loss trials involving dietary changes typically encourage daily dietary self-monitoring, as this can help people become more aware of their dietary adherence, and it tends to be associated with weight loss [13]. However, people commonly dislike daily monitoring, and their adherence to it tends to fade over time [14,15]. Thus, we compared daily versus monthly dietary self-monitoring, as a monthly amount may still be able to help participants self-regulate their dietary intake but in a less burdensome manner. To our knowledge, this is the first trial to randomize participants to different frequencies of the same type of dietary self-monitoring.

The second strategy we assessed was mindful eating. We included exercises to increase awareness of the physical, cognitive, and emotional triggers of overeating; the awareness of internal cues that signal hunger, fullness, and taste satisfaction; “surfing” the urges to reduce emotional eating; and the cultivation of healthier alternatives [16,17]. The materials included, for example, a guided mindful eating exercise, a guided mini meditation to try before meals, and a hunger awareness exercise. We also included more general mindfulness topics, including how to respond versus react to situations. These approaches aim to help participants become more aware of their hunger-related bodily sensations, food cravings, and eating triggers, so that they can choose to respond more deliberately. Previous research has shown that mindful eating training helps reduce emotional eating, which is an important barrier for dietary adherence [18,19], and evidence suggests that increased mindful eating is associated with decreased fasting glucose levels in participants of a mindful eating weight loss intervention [20]. However, these strategies require extra time and attention from participants, which could be burdensome.

The third strategy we tested was positive affect skills training, whose goal was to increase the frequency that participants experienced positive emotions. We taught participants skills such as noticing and savoring positive events, gratitude, acts of kindness, positive reappraisal, applying one’s personal strengths, and setting attainable goals [21]. To adhere to any dietary intervention, participants need to effectively cope with life stressors, and according to the revised Stress and Coping Theory [22,23], positive affect can serve as a psychological *time-out* from stress and increase adaptive coping [24-26]. Moreover, interventions that increase the experience of positive affect can reduce depressive symptoms, anxiety, and stress [27], which themselves may decrease treatment adherence [28]. Positive affect training may improve dietary adherence to the prescribed diet, which can be challenging. Dietary adherence, in this setting, requires participants to cope both emotionally and cognitively without using food-based coping strategies that they may have used in the past. For example, participants must (1) follow external instructions about what to eat and when to do so multiple times a day; (2) maintain supplies of appropriate foods in their homes, workplaces, and social gatherings, which requires planning, negotiating with others, and food refusal skills; (3) follow a way of eating that significant others may not follow or support, which can be isolating and frustrating; (4) plan financially for meals and snacks that other household members might reject or could lead to increased financial stress; (5) override any personal preferences for food or food-based rituals that they might have (such as eating chips at a restaurant or eating popcorn at movie theaters), which are not consistent with their new way of eating; and (6) overcome typical urges to eat when stressed or bored. In addition, hedonic theories of behavior propose that people do more of what they enjoy [29], possibly because positive emotional responses to behaviors increase the motivation and nonconscious desire to engage in those behaviors [30,31], so participants may be more likely to engage in an intervention that they enjoy. Similarly, previous research has demonstrated an association between higher eating plan satisfaction rates and adherence [32,33]. However, as with the mindful eating skills, the positive affect skills require extra time and attention from participants, which could be burdensome.

The primary aim of this study was to assess whether 3 supplementary strategies (daily vs monthly dietary self-monitoring, mindful eating, and positive affect skills training) could improve outcomes in this VLC intervention with adults with T2D.

## Methods

### Ethics Approval

The institutional review board at the University of Michigan approved this research (HUM00115537).

### Participants

This study was registered at ClinicalTrials.gov (NCT03037528). We recruited participants between February 4, 2017, and February 28, 2020, and completed data collection by June 4, 2021. We placed advertisements or notices of the research on the web (including Craigslist, University of Michigan’s web-based portal for clinical trials, and ResearchMatch) and sent invitation letters to potentially eligible participants identified from the health plan records at Michigan Medicine. Interested prospective participants were directed to the study website, which contained the University of Michigan logo, pertinent study information, and a link to a web-based self-reporting screening survey (Qualtrics). Those who were eligible for further screening based on their survey responses were asked to provide web-based electronic consent for the trial and subsequently to complete a second web-based survey (Qualtrics) that included the 8-item Patient Health Questionnaire (PHQ-8) to measure depressive symptoms [34]; a fingerstick self-collected mail-in blood test kit for hemoglobin A_1c_ (HbA_1c_) test from DTI Laboratories, Inc, a Clinical Laboratory Improvement Amendments Certified Reference Laboratory [35]; and 3 days of dietary self-monitoring [36]. We also mailed participants a body weight scale that was connected to their own cellular network (BodyTrace).

### Eligibility Criteria

Prospective participants were invited to enroll if they were aged 21 to 70 years, had a baseline HbA_1c_ of ≥6.5%, had a BMI of 25-45 kg/m^2^ (based on self-reported height and measured weight from the study-provided scale), had regular access to the internet, were willing to check their email at least once a week, were comfortable reading and writing in English, had no potentially serious comorbidities such as liver or kidney failure, were planning on living in the United States for the duration of the trial, were not vegetarian or vegan, were not on weight loss medications, and were not taking warfarin or lithium. We also excluded people who were pregnant or breastfeeding, had an untreated thyroid condition, had an untreated mental health condition, had undergone weight loss surgery in the previous year, or were undergoing cancer treatments. Given that this study was conducted remotely, to mitigate the risk of hypoglycemia, we excluded participants who reported taking any antiglycemic medications other than metformin. Participants who met all the eligibility criteria following the screening process were invited to participate in the trial. They consented using an approved web-based consent form that described the study procedures and goals.

### Trial Design

This 2×2×2 full factorial experimental design examined the impact of 3 experimental, 2-level supplementary strategies. The factorial trial design allows the entire trial population to be used to assess the effects of each factor on the outcome, allowing the efficient assessment of multiple behavioral factors in a single trial [37]. In this design, analysis is conducted to estimate the main effect of each factor by comparing outcomes between combinations of experimental conditions that match, except for the factor whose main effect is being estimated, and combining results across each set of matching combinations, for example, the main effect of including positive affect skills is essentially the difference between conditions (1 – 2) + (3 – 4) + (5 – 6) + (7 – 8; Table 1). Once all baseline measurements had been completed, the study staff randomized the participants to 1 of the 8 combinations of experimental conditions (Table 1) using a computer program to reveal the next assignment. The order was created using block randomization procedures, with blocks randomly allocated to size 8 or 16 and with the seed numbers used for randomization of 64655102233242, 64655183677600 from the Sealed Envelope website [38]. We stratified the randomization by gender.

Some participants were amid their participation in the trial when the COVID-19 outbreak occurred in the United States (30/112, 26.8%). As the intervention was already completely remote, we were able to continue with the trial; however, this may have affected the outcomes.

**Table 1:** Experimental conditions and levels of experimental supplemental strategies.

Experimental condition	Core intervention	Daily dietary self-monitoring frequency	Mindful eating	Positive affect skills
1	Yes	Yes or daily	Yes	Yes
2	Yes	Yes or daily	Yes	No
3	Yes	Yes or daily	No	Yes
4	Yes	Yes or daily	No	No
5	Yes	No or monthly	Yes	Yes
6	Yes	No or monthly	Yes	No
7	Yes	No or monthly	No	Yes
8	Yes	No or monthly	No	No

### Core Intervention

Once participants were assigned to the different intervention strategies, we emailed all participants links to the core intervention on the web, primarily educational VLC intervention materials throughout the 12-month intervention, weekly for the first 4 months, and then every 2 weeks for the remaining 8 months, for a total of 32 emails. Each of the 32 sets of materials focused on a different topic related to following a VLC diet. The emailed links connected participants to (1) a short survey to assess intervention-related dietary adherence and any health concerns; (2) a short, embedded video teaching session topic (eg, managing their diet during holidays or shifting particular meals to be VLC); (3) downloadable handouts to accompany the video; and (4) links to external web-based resources supporting session topics. Transcripts of the embedded videos were also provided.

All participants received the same core, web-based nutritional intervention. This taught participants to eat a VLC diet based on our previous protocol [12], which aimed to limit carbohydrate intake to between 20 and 35 nonfiber grams of carbohydrates per day with the goal of achieving nutritional ketosis. A positive urine dipstick (Bayer Ketostix, which measures ketone acetoacetate) was used as an indicator of nutritional ketosis. Participants were advised to follow a diet that included meat, fish, cheese, eggs, fats, nuts, seeds, and low-carbohydrate vegetables and eliminated starchy and sugary foods. Participants also had email access to a dietary coach (either author KR or MP), as coaches have generally been found to be effective additions to behavioral interventions [39]. Both coaches had extensive experience with the VLC diet, and all messages were checked for fidelity by the first author, LRS, before being sent. Whenever the participants emailed questions to the coaches, they would receive prompt responses with support and resources. Overall, the coaches emailed participants a minimum of every 2 weeks. The participants also received a body weight scale at the start of their participation, and we asked the participants to monitor their body weight regularly, aiming for weighing themselves at least weekly. Coaches used this information to monitor participant success and tailor support. Starting from week 6 of the intervention, we provided goals for physical activity and sleep. Using the Diabetes Prevention Program [40] as a guide, we encouraged participants to be physically active for at least 150 minutes per week. We also encouraged participants to target 7-9 hours of total sleep per day. To encourage the adoption and maintenance of the new intervention-related behaviors, we sent participants text messages up to 5 times a week about the targeted behaviors and skills, depending on which supplemental strategies they were randomized to, as reminders about targeted behaviors are tied to greater behavioral adherence [41]. To help participants change their dietary patterns, we mailed the following cookbooks to participants: *Keto Living 3 Cookbook: Lose Weight with 101 All New Delicious and Low Carb Ketogenic Recipes* [42] at baseline; *Bacon & Butter, the Ultimate Ketogenic Diet Cookbook* [43] at month 3; *The Wicked Good Ketogenic Diet Cookbook: Easy, Whole Food Keto Recipes for Any Budget* [44] at month 6; and *The Everyday Ketogenic Kitchen With More Than 150 Inspirational Low*-*Carb, High*-*Fat Recipes to Maximize Your Health* [45] at month 10. As an incentive for continued participation, we paid participants US $25 for completing their outcome measurements at 4 months, US $25 at 8 months, and US $50 at 12 months.

### Experimental Supplemental Strategies

#### Overview

We randomized the participants to receive a VLC diet and 1 of the 8 possible combinations of the 3 supplemental strategies: dietary self-monitoring, mindful eating, or positive affect skills. The program materials were modified for each supplemental strategy, including different content added to the videos, handouts, and text messages. Participants were aware of the study design, but we did not explicitly state that participants were in the on or off group of each behavioral strategy.

#### Dietary Self-monitoring

All participants were asked to self-monitor their diet using the free web-based or mobile app MyFitnessPal [46], which has a wide variety of foods in its database that are common to the diet assigned in this trial (reducing participant burden and increasing accuracy). Participants were randomized to track their diet either daily or monthly (defined as 3 days every 4 weeks).

#### Mindful Eating Skills

Roughly half of the participants were randomized to training in mindful eating, how to practice the skills in everyday life, research supporting the use of the skills, how and why the skills were expected to help, and targeted suggestions for practicing these skills. We asked participants to focus on consciously savoring their food; eating more slowly; and noticing the textures, flavors, and aromas of their food more carefully. For example, during 1 session, we asked them to practice slowly savoring their food with a snack and encouraged them to practice this skill for at least 1 meal per day over the following week. Multimedia Appendix 1 provides an example handout [47-49].

#### Positive Affect Skills

Roughly half of the participants were randomized to receive training in positive affect skills. They were taught how to practice these skills in everyday life, informed about research supporting the use of the skills, how and why the skills were expected to help, and provided with targeted suggestions for practicing the skills. The skills we taught included noticing and savoring positive events, gratitude, positive reappraisal, and setting attainable goals, similar to our previous research [21]. Multimedia Appendix 1 provides an example handout.

### Assessments

We conducted the following assessments for 1 primary outcome (HbA_1c_), 2 secondary outcomes (weight and depressive symptoms), and several exploratory outcomes (sessions viewed, dietary adherence, intervention satisfaction, metformin use, and qualitative feedback).

#### Glycemic Control

We measured our primary outcome, change in glycemic control at 12 months from baseline, with an at-home HbA_1c_ kit (DTI Laboratories, Inc). The company was masked to the intervention design.

#### Weight

We assessed 1 of our secondary outcomes, change in percent body weight at 12 months from baseline, using the scale we had mailed to participants (BodyTrace). The company was blinded to the intervention design. We asked the participants to stand on the scale twice in 5 minutes at each period, and we used the average of these 2 measurements as their recorded weight.

#### Depressive Symptoms

We assessed 1 of our secondary outcomes, change in depressive symptoms at 12 months from baseline, using the PHQ-8 [34]. This was part of a web-based survey (Qualtrics), which was masked to the intervention design.

#### Sessions Viewed

We tracked the total number of sessions that were viewed by participants.

#### Dietary Adherence

With each session, we asked whether, based on self-assessment, participants were following a VLC diet rated from 1=“not at all” to 7=“very much so,” and we used the average of these ratings as an indicator of self-reported dietary adherence.

#### Intervention Satisfaction

At month 12, we asked participants, “How would you rate your overall satisfaction with the program?” (response options ranged from 1=“not at all satisfied” to 7=“very satisfied”), similar to previous research [50]. This was part of a web-based survey (Qualtrics).

#### Metformin Use

We considered a participant to have changed their metformin dose at 12 months if they reported such a change in their surveys, using the most recent description of the changes as their outcome in the trial.

#### Qualitative Feedback

We asked participants, using open-ended survey questions, about their thoughts about the intervention itself, their health changes, and the responses they had previously received from their physicians. The questions included, “Do have any overall suggestions for improving anything about the program or study?,” “Do have any suggestions about how we can improve how and how much people track what they are eating?”, “Please describe any changes in your medical issues since starting the study. Have they gotten better or worse since starting the study?” and “Have you gotten any feedback from family, friends, or physicians/health care providers about this program or your experience with it? If so, what did they say?”

### Statistical Analysis

To assess the 12-month intervention changes, we conducted complete case analyses, excluding participants who did not complete the relevant 12-month assessment. Collapsing across all participants, we examined pre-post changes in HbA_1c_ (our primary outcome), percent weight, PHQ-8, number of sessions participants viewed, self-reported dietary adherence, and intervention satisfaction, using between subjects 2-tailed *t* tests with SPSS (version 28.0; IBM Corp). We explored the outcomes using intent-to-treat analyses (n=112) with linear mixed regression models using SPSS. This approach makes use of all available data at the baseline and 12-month time points, with the outcomes as dependent variables, time (pre and post), and all 3 strategies in a full factorial design. We explored the overall changes across strategies using within-subjects *t* tests with SPSS. For the qualitative results, we examined open-ended comments and summarized common themes and exemplar quotes.

Sample size calculations were performed using an SAS macro written by Dziak et al [51], designed for factorial trials. Our goal was to screen for supplemental strategies that had at least a medium effect size (Cohen *d*=0.5; Cohen suggested that a small effect size was 0.2, a medium effect size was 0.5, and a large effect size was 0.8). In addition, we planned to explore whether HbA_1c_ and weight changed by a clinically meaningful amount: a reduction by a unit of 0.5% for HbA_1c_ [52] and 3% to 5% reduction in weight [53]. The sample size was also estimated using a desired power of 0.8, a 2-tailed *P* value of .05, and a pretest-posttest correlation of HbA_1c_ (the primary outcome of interest) of between 0.30 and 0.40 (based on pilot data). This led to a sample size goal of 108 to 117 of analyzable cases, which, with a planned attrition rate of 20%, would be 130 to 140 randomized participants. Therefore, our final sample size of 112 was slightly underpowered, after attrition, to detect changes between strategies based on our sample size estimates.

## Results

**Figure 1:**
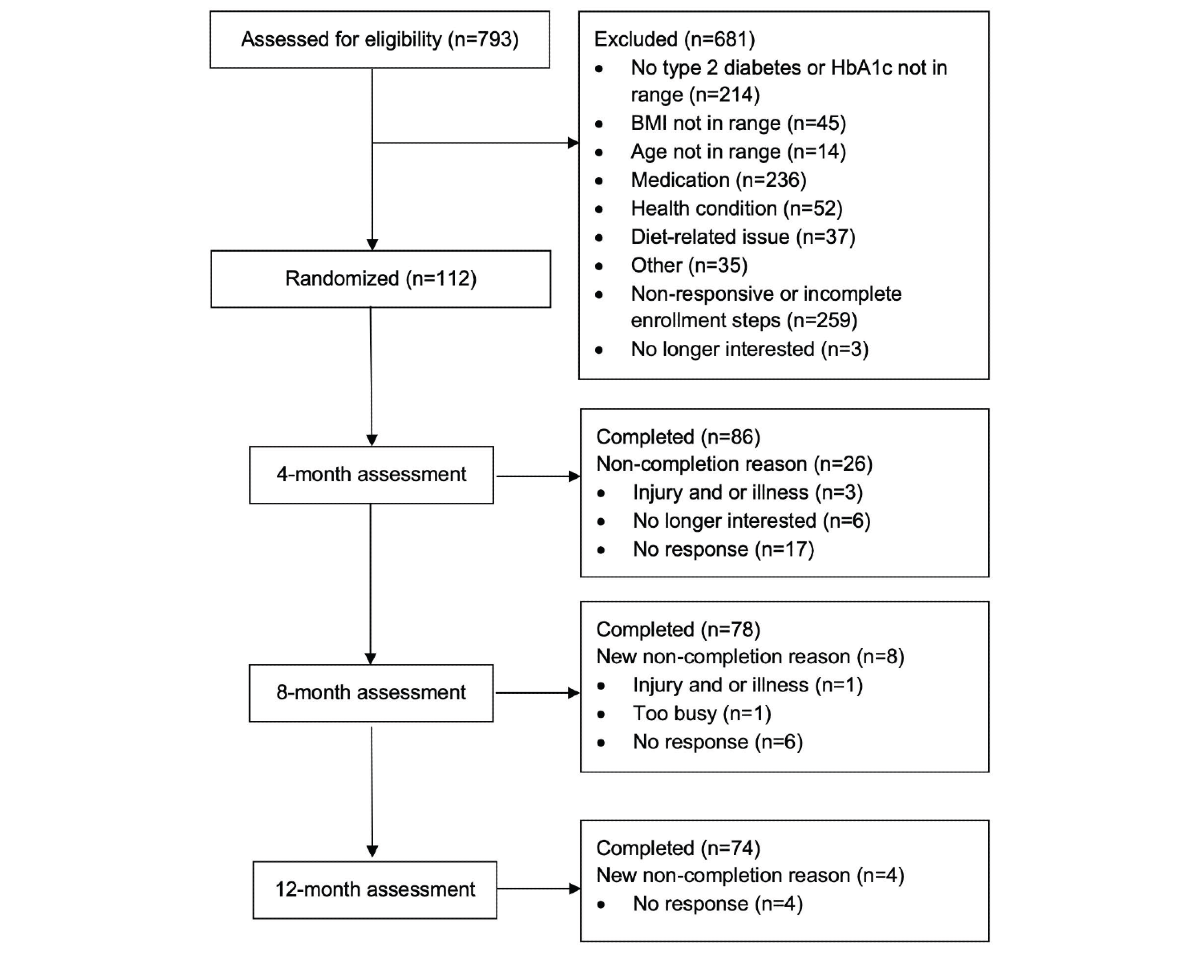
A CONSORT (Consolidated Standards of Reporting Trials) diagram depicting participant flow through the study. HbA_1c_: hemoglobin A_1c_.

We screened 793 potential participants, 112 (14.1%) of whom were randomized (Figure 1). Common reasons for exclusion included being nonresponsive or not completing enrollment steps (n=259), taking an excluded medication such as a nonmetformin antiglycemic medication (n=236), not having T2D or not having an HbA_1c_ measurement in range (n=214), a diet-related issue such as already following a VLC diet (n=37), having an excluded health condition such as an untreated mental illness (n=52), or some other issues such as participating in another study already (n=35). At baseline, participants were on average aged 54.1 (SD 9.6) years, with a BMI of 35.0 (SD 5.1) kg/m^2^ and an HbA_1c_ level of 7.5% (SD 1.1%), and 22.7% (25/110) of the participants had elevated depressive symptoms at baseline according to the PHQ-8. Most patients were White (81/112, 72.3%) and non-Hispanic (108/112, 96.4%; Table 2). We also compared the participants who dropped out and those who did not for these baseline variables (Table 2). No serious adverse events were reported during the trial.

**Table 2:** Baseline participant characteristics by strategy level (N=112).

Experimental supplemental strategy			Age (years), mean (SD)		Gender (female), n (%)		Hemoglobin A_1c_ (%), mean (SD)		BMI (kg/m^2^), mean (SD)		Depressed, n (%)		Race, n (%)							Ethnicity, n (%)		
													White		AA^a^		Other		Hispanic			Not Hispanic
Overall (n=112)			54.1 (9.6)		80 (71.4)		7.5 (1.1)		35.0 (5.1)		61 (54.5)		81 (72.3)		25 (22.3)		7 (6.3)		3 (2.7)			108 (96.4)
**Daily dietary self-monitoring**																						
	Yes (n=57)	52.3 (10.3)		41 (71.9)		7.3 (0.9)		35.5 (5.2)		29 (50.9)		42 (73.7)		13 (22.8)		3 (5.3)		1 (1.8)			56 (98.2)	
	No (n=55)	55.9 (8.7)		39 (70.9)		7.7 (1.2)		34.7 (5.1)		32 (58.2)		39 (70.9)		12 (21.8)		4 (7.3)		2 (3.6)			52 (94.5)	
**Mindful eating**																						
	Yes (n=57)	53.1 (9.2)		40 (70.2)		7.5 (1.2)		35.0 (5.5)		32 (56.1)		44 (77.2)		10 (17.5)		4 (7.0)		1 (1.8)			56 (98.2)	
	No (n=55)	55.0 (10.1)		40 (72.7)		7.5 (1.0)		35.1 (4.8)		29 (52.7)		37 (67.3)		15 (27.2)		3 (5.5)		2 (3.6)			52 (94.5)	
**Positive affect**																						
	Yes (n=56)	54.7 (9.2)		41 (73.2)		7.4 (1.1)		35.7 (5.3)		30 (53.6)		37 (66.1)		14 (25.0)		5 (8.9)		0 (0.0)			55 (98.2)	
	No (n=56)	53.4 (10.1)		41 (73.2)		7.6 (1.1)		34.4 (4.9)		31 (55.4)		44 (78.6)		11 (19.6)		2 (3.6)		3 (5.4)			53 (94.6)	
**Dropout**																						
	Yes (n=38)	53.5 (10.7)		28 (73.7)		7.7 (1.3)		34.9 (5.6)		11 (28.9)		26 (68.4)		10 (26.3)		2 (5.3)		2 (5.3)			36 (94.7)	
	No (n=73)	54.4 (9.1)		54 (73.9)		7.4 (1.0)		35.0 (4.9)		14 (19.2)		55 (75.3)		17 (23.3)		1 (1.4)		1 (1.4)			71 (97.3)	

^a^AA: African American.

The gender of 1 participant was unknown. Participants could specify more than 1 race, and 1 person’s ethnicity was unknown.

Table 3 summarizes the outcomes at 12 months for the complete case analyses, for which no results reached statistical significance and only 1 Cohen *d* point estimate reached 0.5: daily versus monthly dietary self-monitoring had a worse impact on change in depressive symptoms. For our primary outcome, change in HbA_1c_, the 95% CI overlapped with negligible benefit to significant harm for daily (vs monthly) dietary self-monitoring (−0.28% to 1.12%), with significant benefit to negligible harm for mindful eating (−1.15% to 0.22%), and with moderate benefit or harm for positive affect (−0.57% to 0.82%). Other results for daily (vs monthly) dietary self-monitoring were mixed, suggesting an increase in weight (0.98%) and depressive symptoms (Cohen *d*=0.47), less intervention satisfaction (Cohen *d*=−0.20), more sessions viewed (3.02), and greater dietary adherence (Cohen *d*=0.24). For mindful eating, the results suggested a benefit for dietary adherence (Cohen *d*=0.24) and intervention satisfaction (Cohen *d*=0.30). For positive affect, the results suggested a benefit for depressive symptoms (Cohen *d*=−0.32), the number of sessions viewed (3.68), dietary adherence (Cohen *d*=0.16), and intervention satisfaction (Cohen *d*=0.25).

In the linear mixed models, the results of regressions predicting HbA_1c_, weight, and depressive symptoms were similar, and none of the factor × time interactions statistically significantly predicted outcomes (Multimedia Appendix 2).

We also explored overall changes across the groups. In the complete case analyses, across all participants, HbA_1c_ (−0.70%, 95% CI −1.04% to −0.35%; *P*<.001) and percent weight (−6.82, 95% CI −8.57 to −5.08; *P*<.001) improved by a clinically meaningful amount, and PHQ-8 dropped by a Cohen *d* with a medium effect size (−0.67, 95% CI −.92 to −.41; *P*<.001). Overall, 74.1% (83/112) of the participants were taking metformin at baseline. Although we made no medication management recommendations in the trial, by month 12, 30% (25/83) of the participants reduced or discontinued their metformin. None of the participants increased their antiglycemic medications.

**Table 3:** Changes in outcomes from baseline to 12 months using complete case analyses^a^.

Outcome and strategy			Yes, mean change (95% CI)		No, mean change (95% CI)		Yes versus no, mean difference (95% CI)	Yes versus no, Cohen *d* (95% CI)		*P* value
**Change in hemoglobin A_1c_ (%)**										
	Daily dietary self-monitoring (yes or daily, n=45; no or monthly, n=29)	−0.53 (−0.96 to −0.11)		−0.95 (−1.55 to −0.35)		0.42 (−0.28 to 1.12)		0.28 (−0.19 to 0.75)	.24	
	Mindful eating (yes, n=39; no, n=35)	−0.92 (−1.43 to −0.40)		−0.45 (−0.90 to 0.00)		−0.47 (−1.15 to 0.22)		−0.32 (−0.78 to 0.14)	.18	
	Positive affect (yes, n=40; no, n=34)	−0.64 (−1.20 to −0.08)		−0.76 (−1.14 to −0.39)		0.12 (−0.57 to 0.82)		0.08 (−0.37 to 0.54)	.72	
**Change in percent body weight**										
	Daily dietary self-monitoring (yes/daily, n=47; no/monthly, n=30)	−6.45 (−8.78 to −4.11)		−7.42 (−10.15 to −4.69)		0.98 (−2.61 to 4.57)		0.13 (−0.33 to 0.59)	.59	
	Mindful eating (yes, n=40; no, n=37)	−7.22 (−9.42 to −5.02)		−6.40 (−9.26 to −3.55)		−0.81 (−4.32 to 2.70)		−0.11 (−0.56 to 0.34)	.65	
	Positive affect (yes, n=41; no, n=36)	−7.06 (−9.84 to −4.28)		−6.56 (−8.68 to −4.44)		−0.50 (−4.02 to 3.01)		−0.07 (−0.51 to 0.38)	.78	
**Change in depressive symptoms (PHQ-8^b^)**										
	Daily dietary self-monitoring (yes or daily, n=43; no or monthly, n=27)	−2.26 (−3.61 to −0.91)		−4.33 (−6.13 to −2.53)		2.08 (−0.13 to 4.29)		0.47 (−0.02 to 0.95)	.06	
	Mindful eating (yes, n=37; no, n=33)	−2.97 (−4.07 to −1.88)		−3.15 (−5.16 to −1.14)		0.18 (−2.00 to 2.36)		0.04 (−0.43 to 0.51)	.87	
	Positive affect (yes, n=37; no, n=33)	−3.73 (−5.51 to −1.95)		−2.30 (−3.48 to −1.12)		−1.43 (−3.58 to 0.73)		−0.32 (−0.79 to 0.16)	.19	
**Number of sessions viewed**										
	Daily dietary self-monitoring (yes or daily, n=57; no or monthly, n=55)	21.93 (19.30 to 24.56)		18.91 (15.75 to 22.07)		3.02 (−1.03 to 7.08)		0.28 (−0.09 to 0.65)	.14	
	Mindful eating (yes, n=57; no, n=55)	20.46 (17.49 to 23.38)		20.44 (17.55 to 23.37)		0.02 (−4.07 to 4.11)		0.02 (−0.36 to 0.37)	.99	
	Positive affect (yes, n=56; no, n=56)	22.29 (19.53 to 25.04)		18.61 (15.60 to 21.62)		3.68 (−0.36 to 7.71)		.34 (−0.03 to 0.71)	.07	
**Self-reported dietary adherence**										
	Daily dietary self-monitoring (yes or daily, n=56; no or monthly, n=53)	4.87 (4.50 to 5.24)		4.51 (4.05 to 4.97)		0.36 (−0.22 to 0.94)		0.24 (−0.14 to 0.61)	.22	
	Mindful eating (yes, n=55; no, n=54)	4.87 (4.48 to 5.27)		4.51 (4.07 to 4.94)		0.37 (−0.21 to 0.95)		0.24 (−0.13 to 0.62)	.21	
	Positive affect (yes, n=55; no, n=54)	4.82 (4.40 to 5.23)		4.56 (4.14 to 4.98)		0.25 (−0.33 to 0.84)		0.16 (−0.21 to 0.54)	.39	
**12-month intervention satisfaction**										
	Daily dietary self-monitoring (yes or daily, n=44; no or monthly, n=28)	6.07 (5.66 to 6.48)		6.32 (5.91 to 6.73)		−0.25 (−0.86 to 0.35)		−0.20 (−0.68 to 0.27)	.40	
	Mindful eating (yes, n=38; no, n=34)	6.34 (5.99 to 6.69)		5.97 (5.48 to 6.46)		0.37 (−0.21 to 0.96)		0.30 (−0.16 to 0.76)	.21	
	Positive affect (yes, n=38; no, n=34)	6.32 (5.99 to 6.64)		6.00 (5.48 to 6.52)		0.32 (−0.29 to 0.92)		0.25 (−0.21 to 0.72)	.29	

^a^Results from 2-sided *t* tests and *P* values are for the mean differences between receiving and not receiving the behavioral factor.

^b^PHQ-8: 8-item Patient Health Questionnaire.

We explored the participants’ perceptions of the 3 supplemental strategies using open-ended questions on the self-report survey at months 4, 8, and 12. Some participants had a positive experience with dietary self-monitoring, finding it to be helpful (“Tracking forced me to measure and that was an eye-opener at times.”). Others noted that it was difficult to motivate themselves to self-monitor (“It is so easy to eat too much when you don’t keep track, but for some reason I drag my heels on doing it.”), they avoided dietary self-monitoring when they ate food off of the assigned dietary approach (“I actually found myself tracking less especially when I know that I had gone over the carb limit—even a little.”), and reported that it could be difficult to use the app (“I still find MyFitnessPal hard to use. I ended up making my own list of foods I ate and still eat and refer to it often.”). Some participants felt daily tracking was tedious to do (“I hate, hate, hate the tediousness of inputting every freaking tablespoon of butter or every 1/4 cup of half and half or every miniscule morsel that goes into my mouth every day. So, I only actually input that info once a week or so.”), or found it was easy to forget to do (“...I forget about doing it [tracking] and then I forget what I have eaten.”). Participants also felt it was unnecessary to do once they understood what to eat (“I was really good at it in the beginning but once I felt I knew what to do, it just felt too time consuming.”), and it was challenging to keep up long term (“I tracked very well for the first 4 months and then burned out.”). Participants recommended that dietary self-monitoring a couple of times a week would have been ideal. For example, for those in the daily dietary self-monitoring condition, daily self-monitoring may have been too often. One person in the daily self-monitoring condition suggested the following:

I would suggest tracking no more than 3 times a week. Then it wouldn’t be so time consuming.

For those in the monthly self-monitoring condition, self-monitoring only monthly may not have been sufficient. Someone else in the monthly self-monitoring condition suggested the following:

If you tell me I have to track I will, if it’s my choice I won’t. So, [ask participants] to track 1-2 days a week, every week.

The participants also commented on the mindful eating and positive affect skills. Participants who were assigned to both mindful eating and positive affect skills noted the following:

I liked the other parts of this program. Breathing, self-meditation, doing good for others.

The psychological challenges were good, but I was already familiar with most of them.

One participant who received mindful eating and positive affect skills noted that the text messages about those skills were “...quite annoying. Personally, I don’t need that type of encouragement.” Thoughts were specifically mixed regarding mindful eating. One participant described the following:

My least favorite subject was the whole mindful eating thing—a bit squishy for me. I’m sure some people love it, but just not my cup of tea. I just can’t see getting so connected with the process. However, I have started eating like a European—slows you down a bit.

One person noted, “Mindfulness was valuable to me.” Another person assigned to receive mindful eating text messages noted the following:

Sometimes if I feel like cheating, I’d get a text. It was great to get a reminder and encouragement not to give in to the sweets and to stick with what I am doing. It’s like you are all out there rooting me on every day, having a personal cheerleader through text messages.

Another participant noted the following:

The support and psychological skills have made me want to try to stick with it, just to get those benefits.

We examined the qualitative comments regarding other changes and their impacts. Other notable health and medication changes were described by participants:

I no longer take Metformin XR, tums/Rolaids, or the anti-inflammatory medications I was taking for my Ankylosing Spondylitis.

I was going to need a CPAP [continuous positive airway pressure] machine for sleep apnea, but the sleep apnea is gone!!

Another participant commented the following:

When I was diagnosed with diabetes, I felt kind of hopeless. I imagined a life of more and more medication and complications from diabetes. Now I have hope that there is another way, and I’m very thankful.

Participants noted that the physicians were generally supportive:

My internist was floored by my bloodwork results and weight loss...

My doctor calls me her poster child of diabetes. She can’t believe I’ve made the decision from the moment I was diagnosed to not take the metformin, and instead take the harder path of changing my diet to keep my body under control. She applauds my willpower, but to me, if they told me I had cancer, I’d do whatever it took to live. This disease is no different.

## Discussion

### Principal Findings

This study addressed different ways to improve outcomes in a web-based VLC intervention for adults with T2D by using the Multiphase Optimization Strategy framework to examine which of the 3 potentially helpful supplemental behavioral strategies significantly contributed to reduced HbA_1c_ and other outcomes. Overall, the VLC intervention led to statistically significant improvements in glycemic control, weight loss, and depressive symptoms. However, none of the 3 strategies had a statistically significantly different effect on outcomes, and only 1 Cohen *d* point estimate reached our targeted level of 0.5: daily (vs monthly) dietary self-monitoring, such that greater dietary self-monitoring frequency was associated with increased depressive symptoms.

More specifically, we found mixed results for daily dietary self-monitoring. The 95% CIs suggested that daily dietary self-monitoring may be less effective at reducing HbA_1c_ levels than monthly dietary self-monitoring, which may also be less effective at reducing body weight and improving depressive symptoms but more effective at increasing the number of sessions viewed and increasing self-reported dietary adherence. The results also suggest that daily (vs monthly) dietary self-monitoring had lower intervention satisfaction. Our mixed results regarding dietary self-monitoring were echoed in the qualitative feedback, which suggested that participants found frequent (daily vs monthly) self-monitoring to be useful but burdensome. Similarly, previous qualitative research on participants’ experiences with dietary self-monitoring found comparable complaints, such as the experience of tedium [54]. In a study of dietary self-monitoring with MyFitnessPal, approximately half of the participants experienced increased negative emotions [55], and focus groups have found that people dislike dietary self-monitoring apps [56].

Our results suggest that monthly versus daily dietary self-monitoring may improve participant outcomes. Notably, our results contrast with a recent review, which found that 80% (16/20) of the trials found that the more participants self-monitored their diet, the greater was their weight loss. However, the results of the review were correlational, describing how participants’ behavior was associated with the outcomes [57]. As the review described, “no known published, digital weight loss trials randomized participants to varying frequencies of self-monitoring” [57]. Therefore, to our knowledge, this is the first trial to randomize participants to different frequencies of the same type of dietary self-monitoring.

For mindful eating, for our primary outcome of change in HbA_1c_, the 95% CI overlapped with a significant benefit to negligible harm, and other results suggested a possible moderate benefit for dietary adherence and intervention satisfaction. This aligns with previous research, such as a systematic review finding that in randomized trials, mindfulness-based interventions reduced HbA_1c_ levels [58] and another review finding that mindfulness-based interventions may improve medication adherence [59].

The positive affect skills had no detectable impact on changes in HbA_1c_ levels or percent body weight. However, the 95% CIs for positive affect suggest a moderate benefit for changes in depressive symptoms, number of sessions viewed, dietary adherence, and intervention satisfaction. These results align with previous research, such as the finding that positive affect interventions can reduce depressive symptoms, anxiety, and stress [27], including in adults with T2D [21], and that positive affect has been positively correlated with medication adherence [60]. In observational studies, positive affect was associated with better health outcomes including viral load suppression in people living with HIV [61] and better physical function during recovery from stroke, heart attack, or hip fracture [62]. Positive affect is associated with better health behaviors such as physical activity [63-65] and better medication adherence among people living with chronic illnesses [60]. The Positive Pathways to Health theoretical model [66] posits that interventions that increase positive affect have a host of proximal effects including providing a time-out from stress, encouraging more adaptive coping strategies, broadening attention and cognition, reducing reactivity to daily stress, and enhancing social relationships. These proximal effects, in turn, lead to improved health behaviors and better psychological and physical well-being.

Overall, across all conditions, the core VLC diet intervention for adults with T2D led to improved glycemic control, weight loss, and reductions in antiglycemic medication, replicating previous results in adults with T2D [12,67-70]. Participants’ depressive levels were also reduced, replicating some previous research on a VLC diet in adults with T2D [71], although in 1 of our previous trials, we did not find a statistically significant reduction in depressive symptoms [68].

### Limitations and Strengths

Our study has several limitations and strengths. A limitation of the trial was that 80% of the sample were women, 81% were White, and 96% were non-Hispanic, which limits our ability to understand the impact of these supplementary approaches in a more representative sample. Another limitation of the trial was that 27% of the participants were in the trial when COVID-19 began in the United States in 2020; therefore, our conclusions about which supplementary strategies might be most helpful may have been influenced by this. The supplementary strategies all involve self-control, time, and planning to practice regularly. During the pandemic, some people experienced disruptions in their daily routines, such as increased stress, anxiety, and depressive symptoms; financial stress; and social isolation, all of which may have made practicing the supplemental strategies more challenging. Given budgetary limitations, we did not use a gold standard method of tracking dietary adherence, such as 24-hour dietary recalls, but future research should do so when possible. Moreover, the qualitative section was merely an exploration of open-ended comments and not a rigorously conducted qualitative analysis; therefore, caution should be used when interpreting these results, and future research should use a more rigorous approach. In addition, partly because of COVID-19, we did not recruit our intended number of participants, so the trial was underpowered. Moreover, we could only test a finite set of supplementary strategies; therefore, we cannot generalize our conclusions beyond the strategies tested.

A strength of the trial is its innovation, as it is one of the few trials thus far to conduct such a screening experiment, testing a variety of behavioral components, in a behavioral intervention for adults with T2D with HbA_1c_ as an outcome. Another strength was that we were able to recruit participants nationally, enabling for a more nationally representative sample, as we required no in-person measurements. The web-based nature of the trial allowed participants to continue in the trial, regardless of the COVID-19 social distancing protocols. Moreover, because participants did not have to travel to attend the sessions and the sessions, coaching, and assessments occurred when the participants chose, this made participation more convenient and less costly. Finally, to the best of our knowledge, this is the first trial to randomize participants to different frequencies of the same type of dietary self-monitoring.

### Conclusions

Our study was designed to test whether several supplemental behavioral strategies (daily vs monthly dietary self-monitoring, mindful eating skills, and positive affect skills) could improve glycemic control and other outcomes in adults with T2D who were recommended to follow a VLC diet. Overall, we found that none of the 3 selected supplemental strategies under consideration resulted in statistically significant changes. However, for changes in our primary outcome of 12-month HbA_1c_, the 95% CI overlapped with clinically meaningful improvements in glycemic control for monthly (vs daily) dietary self-monitoring and for mindful eating skills (vs no such skills). The addition of monthly (not daily) dietary self-monitoring, mindful eating, and positive affect skills training did not show a definitive benefit, but it is worth further testing. For example, other future tests could use more intensive versions of the behavioral strategies, such as weekly video-based meetings with a coach who actively guides participants through the strategies, or the strategies could be tested with participants with other health conditions, risk factors, or sociocultural needs. Overall, our results support the use of a VLC diet intervention in adults with T2D.

## Appendix

Multimedia Appendix 1 Example educational materials.

Multimedia Appendix 2 Change in outcomes from baseline to 12 months using intent-to-treat analyses from linear mixed models.

Multimedia Appendix 3 CONSORT-eHEALTH checklist (V 1.6.1).

## Abbreviations

HbA_1c_: hemoglobin A_1c_
PHQ-8: 8-item Patient Health Questionnaire
T2D: type 2 diabetes
VLC: very low–carbohydrate
