# Supplementary material for: Psychological Support Strategies for Adults With Type 2 Diabetes in a Very Low–Carbohydrate Web-Based Program: Randomized Controlled Trial
Source: JMIR Diabetes. 2023 May 11;8:e44295. doi: 10.2196/44295 (PMC10214122; doi:10.2196/44295)
Supplement: Multimedia Appendix 1 [file diabetes_v8i1e44295_app1.pdf]

Noticing "Feel Good" Moments

Noticing "Feel Good" Moments (Overview)

**If you spend your whole life waiting for the storm, you'll never enjoy the sunshine.
~ Morris West**

You can notice positive events.
Even when stressed, we can be aware of the positive events in our lives.

Positive events don't have to be big.
They can be very simple, everyday things like a nice interaction or a good cup of coffee.

They can improve your mood and help you better handle stress.
Even small events can have an impact on your mood and your ability to handle stress. The key is to note when they happen.

This week, be mindful of your "feel good" moments. Sometimes, the simplest pleasures in life are right in front of you, waiting to be noticed.

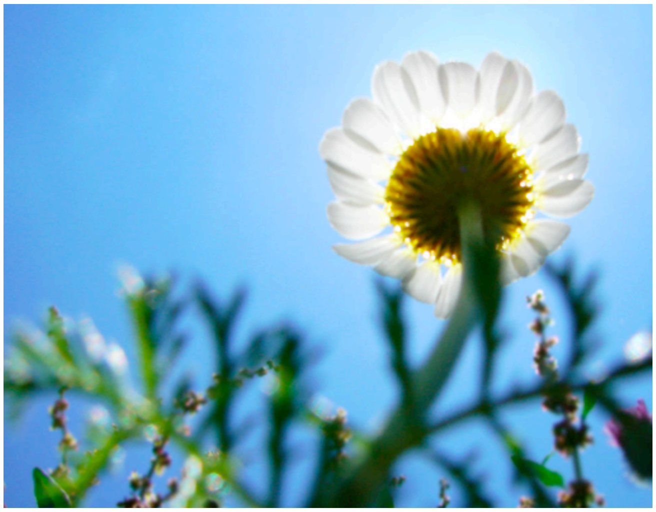

Your weekly challenge: **notice and remember your "feel good" moments**

Each night this week, think about one "feel good" moment from the day. Enjoy the memories. In times of stress this week, try remembering these to help you feel better, calm down, or gain perspective.

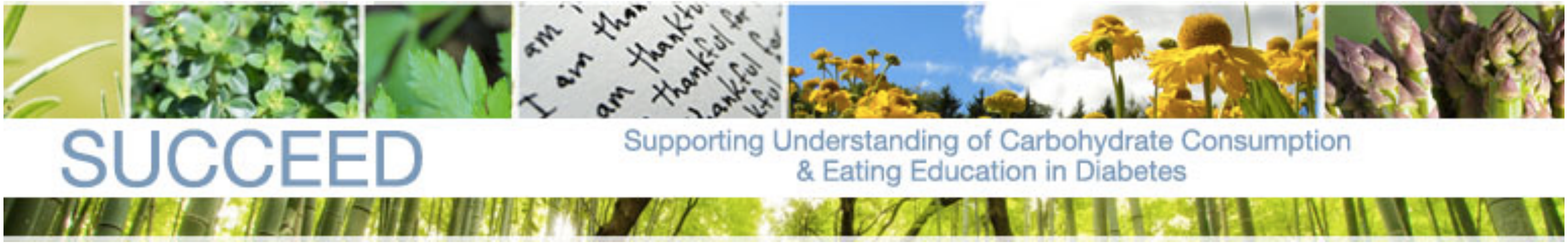

Noticing "Feel Good" Moments

Here is a list of little things to appreciate. Which ones might already be happening to you?

Nice interactions with others:

- good customer service
- someone offers to help
- you were able to give someone directions
- receiving a nice message from a friend
- a stranger smiles at you
- seeing a cute baby
- meeting a friendly dog on the street

Pleasant experiences with nature:

- a pretty view while walking
- a new bloom in the garden
- sweet-smelling flowers
- fresh fruit
- visiting a farmers' market
- seeing the sun rise or set
- appreciating the weather

Other enjoyable daily events:

- wearing comfortable shoes
- getting a short line at the grocery store
- a train that arrives on time
- eating a delicious meal
- coffee just the way you like it

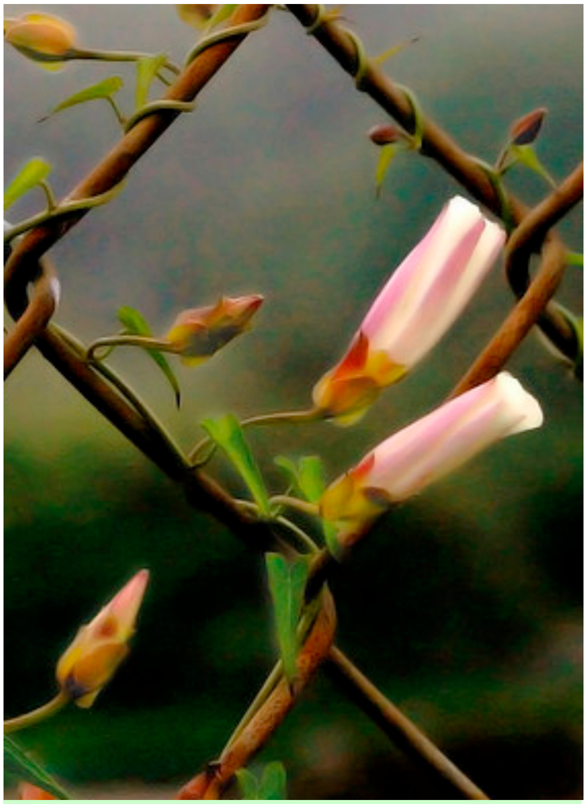

The nicest and sweetest days are not those on which anything very splendid or exciting happens, but those that bring simple little pleasures. ~ L.M. Montgomery

For more ideas of little things to appreciate, check out: http://justlittlethings.net/

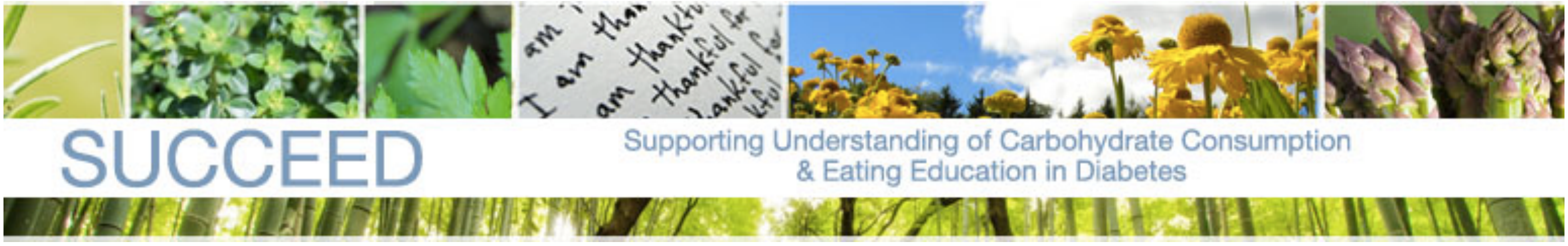

Noticing "Feel Good" Moments

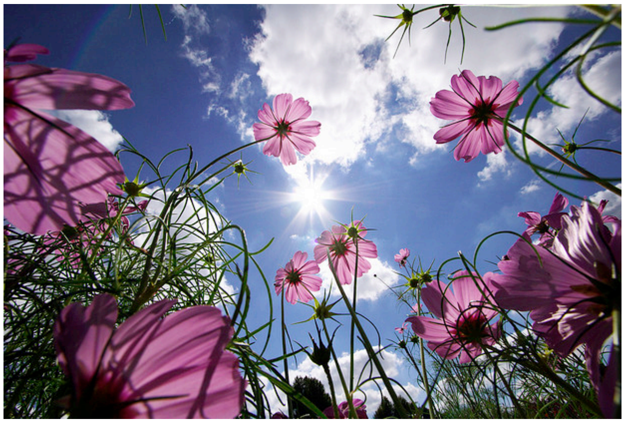

Noticing Positive Events (Some Science)

Many studies show that positive life events are associated with increases in happiness (1, 2). In fact, scheduling "pleasant events" is a central part of some types of therapy for depression (3).

Citations:

1. Zautra AJ & Reich JW (1983) Life events and perceptions of life quality: developments in a two-factor approach. *Journal of Community Psychology* 11:121-132.
2. Murrell SA & Norris FH (1984) Resources, life events, and changes in positive affect and depression in older adults. *American Journal of Community Psychology* 12(4):445-464.
3. Krause N (1998) Positive life events and depressive symptoms in older adults. *Behavioral Medicine* 14 (101-112).

Noticing Positive Events (Even More!)

Check out the Legacy Project, interviews with 1500 elders:
http://legacyproject.human.cornell.edu/category/happiness/
For example:

"A positive attitude depends on thinking small: **the morning cup of coffee, a warm bed on a winter night, a brightly colored bird feeding on the lawn, an unexpected letter from a friend, even a favorite song on the radio.**"

"Ursula suffered immense hardship as a child in Hitler's Germany. Her advice: 'I think the most important thing I learned was not to take things for granted. You cannot be entirely prepared for what will happen to you in life, but I learned that despite everything that happened, life is worth living and **you can enjoy every day**.'"

Happiness from a positive event can provide a momentary break from feelings of stress, unhappiness, or anxiety.

Awareness of Hunger and Fullness

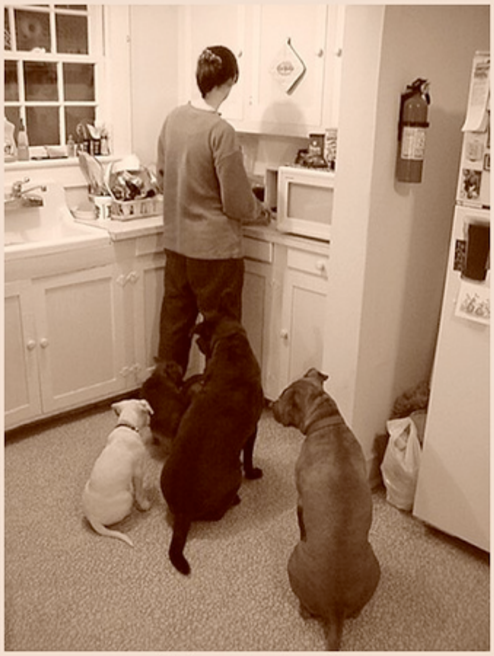

Dinner time!

Mindfulness of Hunger

Sometimes we can overeat because we fail to eat when we are truly hungry. We can learn to become more aware of our hunger.

- How hungry are you right now?:
  As hungry as possible (10) or not at all hungry (1)?
- How does your hunger feel? What sensations do you have?

Types of Hunger

Habitual hunger: Some situations we've learned to associate with eating: at a party, while watching TV, while bored. The body can make us feel hungry when it sees these external cues, but that hunger can go away if we change the situation or distract ourselves.

Stress-based hunger: We can sometimes eat to soothe ourselves from stress. Our stress hormones mobilize our energy, so after being stressed for a bit, we get the signal that we need to replenish that energy. Unless we are truly hungry, this is a false alarm, a drive to eat when we don't actually need to.

Eating for pleasure: We eat because food tastes good. This is normal; our bodies are hard-wired to seek pleasure from food. However, stress can enhance the pleasure value of food, which can prolong eating. Much of the pleasure response is often fleeting, occurring mainly at the beginning. We can notice how our taste satisfaction changes over time and stop eating when we feel satisfied or have had enough of a certain food or flavor.

Reactive hypoglycemia: When our blood sugar dips too low, we may feel shaky, irritable, confused, light headed, or panicky. A way to prevent this is to eat food that doesn't quickly spike (and then drop) your blood sugar. You can also eat more often. Prepare ahead so you will have appropriate snacks and food at the ready!

> **I never planned what I was going to eat until I was hungry, which was like waiting until I was drunk to start driving.**
> **~ Jennette Fulda**

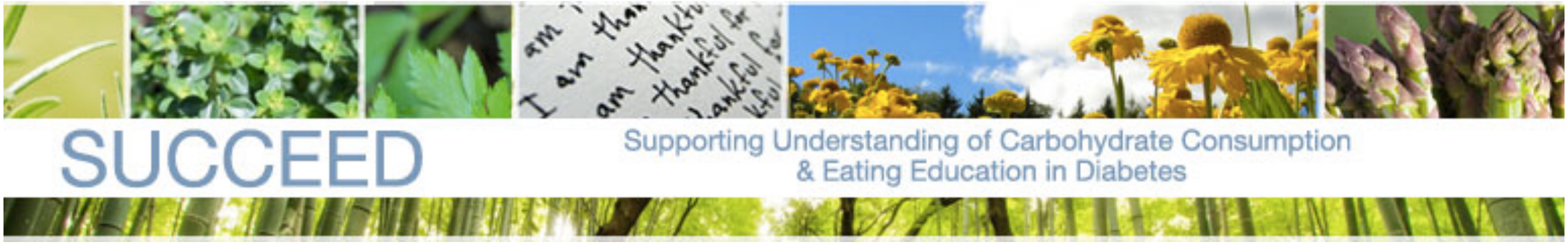

Awareness of Hunger and Fullness

Your weekly challenge: practice **being more mindful of your hunger!**

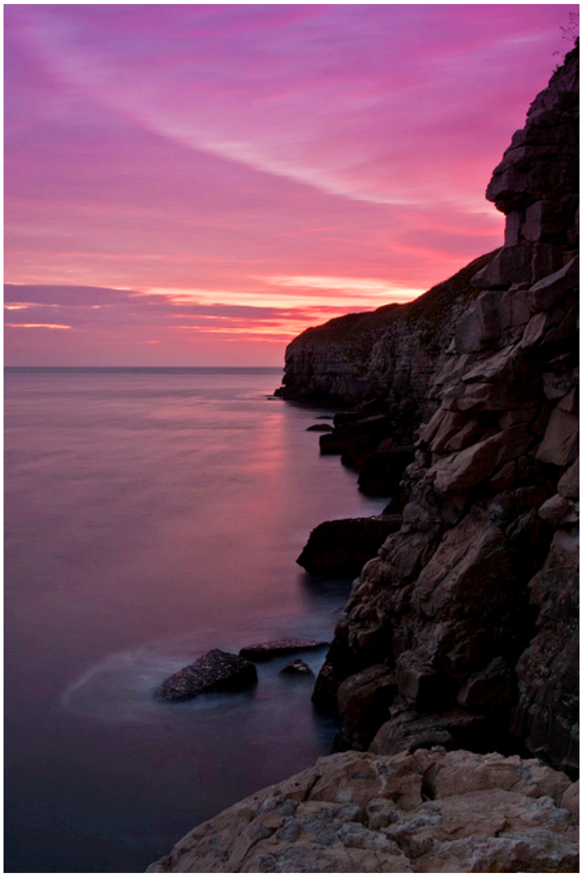

Hunger Awareness (practice)

Bring yourself to a centered place, and bring your attention gently to your breath, closing your eyes as you wish. Follow the breath in and out a few times. Now bring your awareness to your entire body. Notice to yourself right now how hungry you are, with 10 being as hungry as possible, and 1 not being hungry at all… Now that you've found your hunger level, how do you know that? What experiences or sensations helped you find the right number? Now come back to the breath….back to the room… and when you're ready, open your eyes.

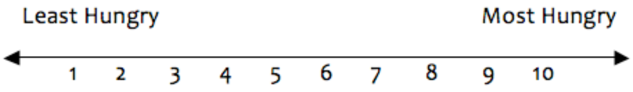

When we don't taste what we eat, we can end up stuffed to the gills but feeling completely unsatisfied. This is because the mind and mouth weren't present, weren't tasting or enjoying, as we ate. The stomach became full but the mind and mouth were unfulfilled and continued calling for us to eat.
~ Jan Chosen Bays

Awareness of Hunger and Fullness

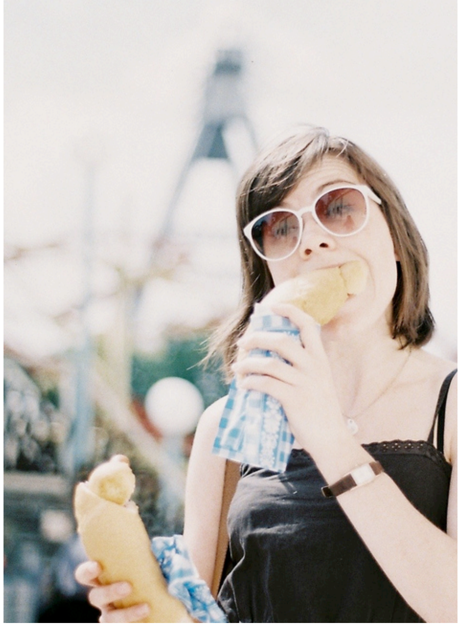

Mindfulness of Fullness

How do we know when to stop eating? We get a variety of signals from inside and outside our bodies.

There are many types of reasons why we decide to stop eating, some from inside our body (fullness or satiety signals) and others from outside our body. We are often unaware of these signals, either ignoring subtle internal signals to stop eating or letting external signals override them. It's important to pay attention to internal cues, the biological signals of satiety.

Types of Fullness

Taste satisfaction (or taste satiety) can kick in early, sometimes after a few bites. Let yourself enjoy and savor the satisfaction from each bite may lead to earlier taste satiety. In other words, with just a little bit of a particular food, you may feel like you have had enough.

Stomach satiety (fullness) kicks in gradually, based on volume of food. Try to notice gradual increases in fullness, before you feel uncomfortable. You might explore how different types of food lead to different feelings of fullness.

Biochemical signals in the body and brain are the slowest satiety signals, and may only kick in after 20 minutes or longer. These signals may not kick in much at all if you eat very sugary foods or if you are tired. Being poorly rested actually alters our hormones of hunger and fullness, making it easier to feel hungry and harder to feel full. Most people need at least 7 hours of sleep a night to be well rested (and to get those hormones working properly).

Your weekly challenge:
eat when hungry and stop when full

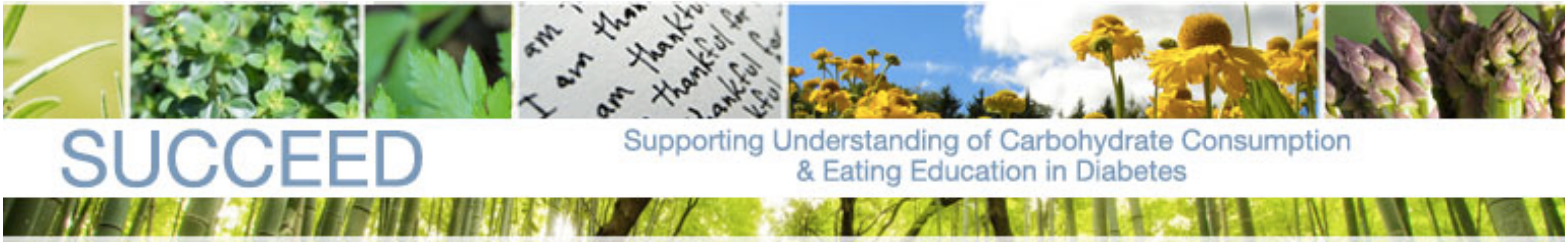

Awareness of Hunger and Fullness

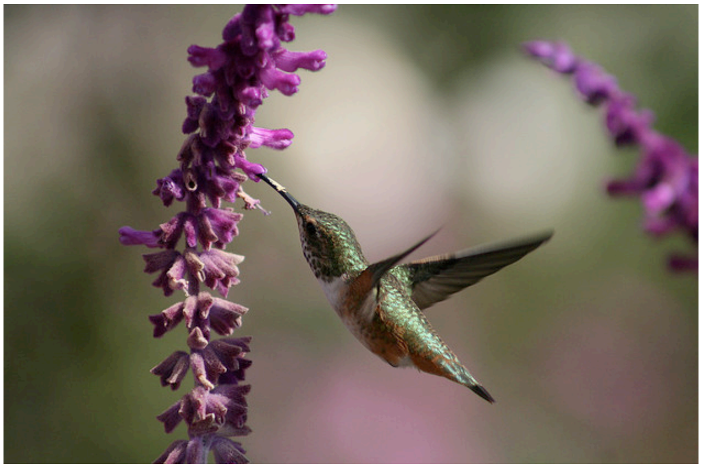

Awareness of Fullness (practice)
Allow your eyes to rest closed for a series of breaths. You can rest your hands on your stomach, and take 6-8 easy, deep breaths… Not forcing the breath, but just inviting it all the way to the bottom of the lungs… You may feel your chest rise and fall… And as the breath gently reaches the bottom of your lungs, you may feel your abdomen expand on the in breath, and contract on the out breath… Just noticing what sensations you have at this time… How physically full are you feeling right now? What is your experience of your stomach? On a scale of 1 – 10, how full are you, right now, with 10 being as full as possible, and 1 being not full at all. How hungry are you? Take a moment to be mindful of all sensations. Now return your attention back to your breath, take a few more deep relaxing breaths, and return your attention to the space of the room.

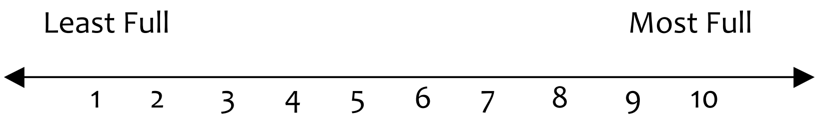

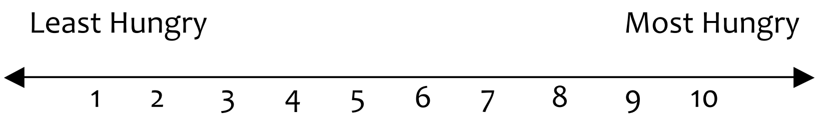

Hunger and fullness are related, but they are not necessarily the same. They are controlled by different parts of the brain and stomach. At first, you may think of them as similar. However, you may also begin to notice differences at certain times. For example, you may be hungry and drink a large glass of water, giving you a sense of fullness in your stomach, but still feel hungry. Or, you may eat a small piece of food and feel less hungry, but your stomach may still be relatively empty.

Thank you to Kjunstorm, flatsworldedge, Muffet, alice_henneman, Tabsinthe, honorthegift, powi, agustinrafaelreyes, calliope, linhngan, and mikebehnken of Flikr for the CC images.
