# Supplementary material for: Psychological Support Strategies for Adults With Type 2 Diabetes in a Very Low–Carbohydrate Web-Based Program: Randomized Controlled Trial
Source: JMIR Diabetes. 2023 May 11;8:e44295. doi: 10.2196/44295 (PMC10214122; doi:10.2196/44295)
Supplement: Multimedia Appendix 2 [file diabetes_v8i1e44295_app2.pdf]

Multimedia Appendix 2. Change in outcomes from baseline to 12 months using intent-to-treat analyses from linear mixed models.

Outcome	Strategy	Yes, mean change (95% CI)	No, mean change (95% CI)	Yes vs. No mean difference	P value
Change in HbA_{1c} (unit of %)					
	Daily Dietary Self–Monitoring (Yes/Daily, No/Monthly)	−0.55 (−0.99 to −0.11)	−1.02 (−1.54 to −0.51)	0.47	0.44
	Mindful Eating (Yes, No)	−0.90 (−1.37 to −0.43)	−0.67 (−1.16 to −0.18)	−0.23	0.84
	Positive Affect (Yes, No)	−0.61 (−1.07 to −0.15)	−0.96 (−1.45 to −0.46)	0.35	0.84
Change in Body Weight (lbs)					
	Daily Dietary Self–Monitoring (Yes, No)	−14.27 (−19.44 to −9.11)	−15.74 (−22.32 to −9.16)	1.47	0.91
	Mindful Eating (Yes, No)	−15.63 (−21.32 to −9.94)	−14.39 (−20.52 to −8.25)	−1.24	0.77
	Positive Affect (Yes, No)	−16.21 (−21.81 to −10.61)	−13.81 (−20.02 to −7.59)	−2.4	0.55
Change in Depressive symptoms (PHQ−8)					
	Daily Dietary Self–Monitoring (Yes, No)	−2.65 (−4.01 to −1.29)	−3.64 (−5.30 to −1.99)	0.99	0.48
	Mindful Eating (Yes, No)	−3.34 (−4.81 to −1.87)	−2.96 (−4.52 to −1.39)	−0.38	0.38
	Positive Affect (Yes, No)	−3.87 (−5.32 to −2.41)	−2.43 (−4.00 to −0.85)	−1.44	0.52

Note: The P values are for the mean differences between receiving and not receiving the behavioral factor.
