# Supplementary material for: Psychological Support Strategies for Adults With Type 2 Diabetes in a Very Low–Carbohydrate Web-Based Program: Randomized Controlled Trial
Source: JMIR Diabetes. 2023 May 11;8:e44295. doi: 10.2196/44295 (PMC10214122; doi:10.2196/44295)
Supplement: Multimedia Appendix 3 [file diabetes_v8i1e44295_app3.pdf]

CONSORT-EHEALTH (V 1.6.1) - Submission/Publication Form

The CONSORT-EHEALTH checklist is intended for authors of randomized trials evaluating web-based and Internet-based applications/interventions, including mobile interventions, electronic games (incl multiplayer games), social media, certain telehealth applications, and other interactive and/or networked electronic applications. Some of the items (e.g. all subitems under item 5 - description of the intervention) may also be applicable for other study designs.

The goal of the CONSORT EHEALTH checklist and guideline is to be
a) a guide for reporting for authors of RCTs,
b) to form a basis for appraisal of an ehealth trial (in terms of validity)

CONSORT-EHEALTH items/subitems are MANDATORY reporting items for studies published in the Journal of Medical Internet Research and other journals / scientific societies endorsing the checklist.

Items numbered 1., 2., 3., 4a., 4b etc are original CONSORT or CONSORT-NPT (non-pharmacologic treatment) items.
Items with Roman numerals (i., ii, iii, iv etc.) are CONSORT-EHEALTH extensions/clarifications.

As the CONSORT-EHEALTH checklist is still considered in a formative stage, we would ask that you also RATE ON A SCALE OF 1-5 how important/useful you feel each item is FOR THE PURPOSE OF THE CHECKLIST and reporting guideline (optional).

Mandatory reporting items are marked with a red *.
In the textboxes, either copy & paste the relevant sections from your manuscript into this form - please include any quotes from your manuscript in QUOTATION MARKS,
or answer directly by providing additional information not in the manuscript, or elaborating on why the item was not relevant for this study.

YOUR ANSWERS WILL BE PUBLISHED AS A SUPPLEMENTARY FILE TO YOUR PUBLICATION IN JMIR AND ARE CONSIDERED PART OF YOUR PUBLICATION (IF ACCEPTED).
Please fill in these questions diligently. Information will not be copyedited, so please use proper spelling and grammar, use correct capitalization, and avoid abbreviations.

DO NOT FORGET TO SAVE AS PDF _AND_ CLICK THE SUBMIT BUTTON SO YOUR ANSWERS ARE IN OUR DATABASE !!!

Citation Suggestion (if you append the pdf as Appendix we suggest to cite this paper in the caption):

Eysenbach G, CONSORT-EHEALTH Group
CONSORT-EHEALTH: Improving and Standardizing Evaluation Reports of Web-based and Mobile Health Interventions
J Med Internet Res 2011;13(4):e126
URL: http://www.jmir.org/2011/4/e126/
doi: 10.2196/jmir.1923
PMID: 22209829

saslowl@umich.edu (not shared) Switch account

Draft saved

* Required

Your name *

First Last

Laura Saslow

Primary Affiliation (short), City, Country *

University of Toronto, Toronto, Canada

University of Michigan, Ann Arbor, MI, USA

Your e-mail address *

abc@gmail.com

saslowl@umich.edu

Title of your manuscript *

Provide the (draft) title of your manuscript.

Psychological Support Strategies for Adults with Type 2 Diabetes in a Very Low–Carbohydrate Web-Based Program: A Randomized Trial

Name of your App/Software/Intervention *

If there is a short and a long/alternate name, write the short name first and add the long name in brackets.

This information is not applicable.

Evaluated Version (if any)

e.g. "V1", "Release 2017-03-01", "Version 2.0.27913"

Your answer

Language(s) *

What language is the intervention/app in? If multiple languages are available, separate by comma (e.g. "English, French")

This information is not applicable.

URL of your Intervention Website or App

e.g. a direct link to the mobile app on app in appstore (itunes, Google Play), or URL of the website. If the intervention is a DVD or hardware, you can also link to an Amazon page.

Your answer

URL of an image/screenshot (optional)

Your answer

Accessibility *

Can an enduser access the intervention presently?

- ( ) access is free and open
- ( ) access only for special usergroups, not open
- ( ) access is open to everyone, but requires payment/subscription/in-app purchases
- (•) app/intervention no longer accessible
- ( ) Other:

Primary Medical Indication/Disease/Condition *

e.g. "Stress", "Diabetes", or define the target group in brackets after the condition, e.g. "Autism (Parents of children with)", "Alzheimers (Informal Caregivers of)"

Type 2 diabetes

Primary Outcomes measured in trial *

comma-separated list of primary outcomes reported in the trial

HbA1c

Secondary/other outcomes

Are there any other outcomes the intervention is expected to affect?

percent body weight, depressive symptoms, dietary adherence, number of classes viewed

Recommended "Dose" *

What do the instructions for users say on how often the app should be used?

- ( ) Approximately Daily
- (•) Approximately Weekly
- ( ) Approximately Monthly
- ( ) Approximately Yearly
- ( ) "as needed"
- ( ) Other:

Approx. Percentage of Users (starters) still using the app as recommended after 3 months *

- ( ) unknown / not evaluated
- ( ) 0-10%
- ( ) 11-20%
- ( ) 21-30%
- ( ) 31-40%
- ( ) 41-50%
- ( ) 51-60%
- (•) 61-70%
- ( ) 71%-80%
- ( ) 81-90%
- ( ) 91-100%
- ( ) Other:

Overall, was the app/intervention effective? *

- ( ) yes: all primary outcomes were significantly better in intervention group vs control
- ( ) partly: SOME primary outcomes were significantly better in intervention group vs control
- (•) no statistically significant difference between control and intervention
- ( ) potentially harmful: control was significantly better than intervention in one or more outcomes
- ( ) inconclusive: more research is needed
- ( ) Other:

Article Preparation Status/Stage *

At which stage in your article preparation are you currently (at the time you fill in this form)

- ( ) not submitted yet - in early draft status
- (•) not submitted yet - in late draft status, just before submission
- ( ) submitted to a journal but not reviewed yet
- ( ) submitted to a journal and after receiving initial reviewer comments
- ( ) submitted to a journal and accepted, but not published yet
- ( ) published
- ( ) Other:

Journal *

If you already know where you will submit this paper (or if it is already submitted), please provide the journal name (if it is not JMIR, provide the journal name under "other")

- ( ) not submitted yet / unclear where I will submit this
- (x) Journal of Medical Internet Research (JMIR)
- ( ) JMIR mHealth and UHealth
- ( ) JMIR Serious Games
- ( ) JMIR Mental Health
- ( ) JMIR Public Health
- ( ) JMIR Formative Research
- ( ) Other JMIR sister journal
- ( ) Other:

Is this a full powered effectiveness trial or a pilot/feasibility trial? *

- (x) Pilot/feasibility
- ( ) Fully powered

Manuscript tracking number *

If this is a JMIR submission, please provide the manuscript tracking number under "other" (The ms tracking number can be found in the submission acknowledgement email, or when you login as author in JMIR. If the paper is already published in JMIR, then the ms tracking number is the four-digit number at the end of the DOI, to be found at the bottom of each published article in JMIR)

- (selected) no ms number (yet) / not (yet) submitted to / published in JMIR
- Other:

TITLE AND ABSTRACT

1a) TITLE: Identification as a randomized trial in the title

1a) Does your paper address CONSORT item 1a? *

I.e does the title contain the phrase "Randomized Controlled Trial"? (if not, explain the reason under "other")

- (selected) yes
- Other:

1a-i) Identify the mode of delivery in the title

Identify the mode of delivery. Preferably use “web-based” and/or “mobile” and/or “electronic game” in the title. Avoid ambiguous terms like “online”, “virtual”, “interactive”. Use “Internet-based” only if Intervention includes non-web-based Internet components (e.g. email), use “computer-based” or “electronic” only if offline products are used. Use “virtual” only in the context of “virtual reality” (3-D worlds). Use “online” only in the context of “online support groups”. Complement or substitute product names with broader terms for the class of products (such as “mobile” or “smart phone” instead of “iphone”), especially if the application runs on different platforms.

subitem not at all important

1 ○

2 ○

3 ◉

4 ○

5 ○

essential

Clear selection

Does your paper address subitem 1a-i? *

Copy and paste relevant sections from manuscript title (include quotes in quotation marks "like this" to indicate direct quotes from your manuscript), or elaborate on this item by providing additional information not in the ms, or briefly explain why the item is not applicable/relevant for your study

Yes, we describe that it is web-based.

1a-ii) Non-web-based components or important co-interventions in title

Mention non-web-based components or important co-interventions in title, if any (e.g., “with telephone support”).

subitem not at all important

1 ○

2 ○

3 ◉

4 ○

5 ○

essential

Clear selection

Does your paper address subitem 1a-ii?

Copy and paste relevant sections from manuscript title (include quotes in quotation marks "like this" to indicate direct quotes from your manuscript), or elaborate on this item by providing additional information not in the ms, or briefly explain why the item is not applicable/relevant for your study

too many other aspects to describe well

1a-iii) Primary condition or target group in the title

Mention primary condition or target group in the title, if any (e.g., “for children with Type I Diabetes”) Example: A Web-based and Mobile Intervention with Telephone Support for Children with Type I Diabetes: Randomized Controlled Trial

subitem not at all important

1 ○

2 ○

3 ○

4 ○

5 ●

essential

Clear selection

Does your paper address subitem 1a-iii? *

Copy and paste relevant sections from manuscript title (include quotes in quotation marks "like this" to indicate direct quotes from your manuscript), or elaborate on this item by providing additional information not in the ms, or briefly explain why the item is not applicable/relevant for your study

"Adults with Type 2 Diabetes"

1b) ABSTRACT: Structured summary of trial design, methods, results, and conclusions

NPT extension: Description of experimental treatment, comparator, care providers, centers, and blinding status.

1b-i) Key features/functionalities/components of the intervention and comparator in the METHODS section of the ABSTRACT

Mention key features/functionalities/components of the intervention and comparator in the abstract. If possible, also mention theories and principles used for designing the site. Keep in mind the needs of systematic reviewers and indexers by including important synonyms. (Note: Only report in the abstract what the main paper is reporting. If this information is missing from the main body of text, consider adding it)

subitem not at all important

1 ○

2 ○

3 ○

4 ◉

5 ○

essential

Clear selection

Does your paper address subitem 1b-i? *

Copy and paste relevant sections from the manuscript abstract (include quotes in quotation marks "like this" to indicate direct quotes from your manuscript), or elaborate on this item by providing additional information not in the ms, or briefly explain why the item is not applicable/relevant for your study

"They received a remotely-delivered 12-month VLC diet intervention. Participants were randomly assigned through a full-factorial 2x2x2 design to supplementary strategies: either daily or monthly dietary self-monitoring, either mindful eating training or not, and either positive affect skills training or not."

1b-ii) Level of human involvement in the METHODS section of the ABSTRACT

Clarify the level of human involvement in the abstract, e.g., use phrases like “fully automated” vs. “therapist/nurse/care provider/physician-assisted” (mention number and expertise of providers involved, if any). (Note: Only report in the abstract what the main paper is reporting. If this information is missing from the main body of text, consider adding it)

subitem not at all important

1 ○

2 ○

3 ◉

4 ○

5 ○

essential

Clear selection

Does your paper address subitem 1b-ii?

Copy and paste relevant sections from the manuscript abstract (include quotes in quotation marks "like this" to indicate direct quotes from your manuscript), or elaborate on this item by providing additional information not in the ms, or briefly explain why the item is not applicable/relevant for your study

not enough space to mention

1b-iii) Open vs. closed, web-based (self-assessment) vs. face-to-face assessments in the METHODS section of the ABSTRACT

Mention how participants were recruited (online vs. offline), e.g., from an open access website or from a clinic or a closed online user group (closed usergroup trial), and clarify if this was a purely web-based trial, or there were face-to-face components (as part of the intervention or for assessment). Clearly say if outcomes were self-assessed through questionnaires (as common in web-based trials). Note: In traditional offline trials, an open trial (open-label trial) is a type of clinical trial in which both the researchers and participants know which treatment is being administered. To avoid confusion, use “blinded” or “unblinded” to indicated the level of blinding instead of “open”, as “open” in web-based trials usually refers to “open access” (i.e. participants can self-enrol). (Note: Only report in the abstract what the main paper is reporting. If this information is missing from the main body of text, consider adding it)

subitem not at all important

1 ( )

2 ( )

3 (•)

4 ( )

5 ( )

essential

Clear selection

Does your paper address subitem 1b-iii?

Copy and paste relevant sections from the manuscript abstract (include quotes in quotation marks "like this" to indicate direct quotes from your manuscript), or elaborate on this item by providing additional information not in the ms, or briefly explain why the item is not applicable/relevant for your study

not enough space to mention

1b-iv) RESULTS section in abstract must contain use data

Report number of participants enrolled/assessed in each group, the use/uptake of the intervention (e.g., attrition/adherence metrics, use over time, number of logins etc.), in addition to primary/secondary outcomes. (Note: Only report in the abstract what the main paper is reporting. If this information is missing from the main body of text, consider adding it)

subitem not at all important

1 ○

2 ○

3 ◉

4 ○

5 ○

essential

Clear selection

Does your paper address subitem 1b-iv?

Copy and paste relevant sections from the manuscript abstract (include quotes in quotation marks "like this" to indicate direct quotes from your manuscript), or elaborate on this item by providing additional information not in the ms, or briefly explain why the item is not applicable/relevant for your study

We mention number of classes viewed: "Other results for daily (vs. monthly) dietary self-monitoring were mixed, suggesting an increase in weight (0.98%) and depressive symptoms (Cohen's d 0.47), less intervention satisfaction (Cohen's d –0.20), more classes viewed (2.05), and greater dietary adherence (Cohen's d 0.24). For mindful eating, results suggested a benefit for dietary adherence (Cohen's d 0.24) and intervention satisfaction (Cohen's d 0.30). For positive affect, results suggested a benefit for depressive symptoms (Cohen's d –0.32), number of classes viewed (2.04), dietary adherence (Cohen's d 0.16), and intervention satisfaction (Cohen's d 0.25)."

1b-v) CONCLUSIONS/DISCUSSION in abstract for negative trials

Conclusions/Discussions in abstract for negative trials: Discuss the primary outcome - if the trial is negative (primary outcome not changed), and the intervention was not used, discuss whether negative results are attributable to lack of uptake and discuss reasons. (Note: Only report in the abstract what the main paper is reporting. If this information is missing from the main body of text, consider adding it)

subitem not at all important

- 1 ○
- 2 ○
- 3 ◉
- 4 ○
- 5 ○

essential

Clear selection

Does your paper address subitem 1b-v?

Copy and paste relevant sections from the manuscript abstract (include quotes in quotation marks "like this" to indicate direct quotes from your manuscript), or elaborate on this item by providing additional information not in the ms, or briefly explain why the item is not applicable/relevant for your study

"The addition of monthly (not daily) dietary self-monitoring, mindful eating, and positive affect skills training did not show definitive benefit but are worth testing further."

INTRODUCTION

2a) In INTRODUCTION: Scientific background and explanation of rationale

2a-i) Problem and the type of system/solution

Describe the problem and the type of system/solution that is object of the study: intended as stand-alone intervention vs. incorporated in broader health care program? Intended for a particular patient population? Goals of the intervention, e.g., being more cost-effective to other interventions, replace or complement other solutions? (Note: Details about the intervention are provided in “Methods” under 5)

subitem not at all important

1 ○

2 ○

3 ○

4 ◉

5 ○

essential

Clear selection

Does your paper address subitem 2a-i? *

Copy and paste relevant sections from the manuscript (include quotes in quotation marks "like this" to indicate direct quotes from your manuscript), or elaborate on this item by providing additional information not in the ms, or briefly explain why the item is not applicable/relevant for your study

"However, long-term adherence to any behavioral intervention can be challenging, and it is not clear which behavioral strategies may be able to help improve outcomes in VLC interventions. In this trial, we screened three potentially effective supplemental behavioral strategies using a full-factorial design....In the current trial, we examined three supplemental strategies that were low-cost and varied in their burdensomeness and level of previous testing. "

2a-ii) Scientific background, rationale: What is known about the (type of) system

Scientific background, rationale: What is known about the (type of) system that is the object of the study (be sure to discuss the use of similar systems for other conditions/diagnoses, if appropiate), motivation for the study, i.e. what are the reasons for and what is the context for this specific study, from which stakeholder viewpoint is the study performed, potential impact of findings [2]. Briefly justify the choice of the comparator.

subitem not at all important

1 ○

2 ○

3 ○

4 ◉

5 ○

essential

Clear selection

Does your paper address subitem 2a-ii? *

Copy and paste relevant sections from the manuscript (include quotes in quotation marks "like this" to indicate direct quotes from your manuscript), or elaborate on this item by providing additional information not in the ms, or briefly explain why the item is not applicable/relevant for your study

"In the current trial, we examined three supplemental strategies that were low-cost and varied in their burdensomeness and level of previous testing. The first strategy we tested was dietary self-monitoring, wherein we varied whether we encouraged participants to practice dietary self-monitoring daily versus monthly (with monthly being defined as monitoring one's diet in bursts of three days every four weeks). Weight loss trials involving dietary changes typically encourage daily dietary self-monitoring, as this can help people to become more aware of their dietary adherence, and it tends to be associated with weight loss [13]. However, people commonly dislike daily monitoring and their adherence to it tends to fade over time [14, 15]. Thus, we compared daily versus monthly dietary self-monitoring, as a monthly amount may still be able to help participants self-regulate their dietary intake, but in a less burdensome manner. To our knowledge, this is the first trial that has randomized participants to differing frequencies of the same type of dietary self-monitoring.

The second strategy we assessed was mindful eating. We included exercises to increase awareness of the physical, cognitive, and emotional triggers of overeating; the awareness of internal cues that signal hunger, fullness, and taste satisfaction; "surfing" the urges to reduce emotional eating; and the cultivation of healthier alternatives [16, 17]. The materials included, for example, a guided mindful eating exercise, a guided mini meditation to try before meals, and a hunger-awareness exercise. We also included more general mindfulness topics, including how to respond versus react to situations. These approaches aim to help participants become more aware of their hunger-related bodily sensations, food cravings, and eating triggers, so that they can choose to respond more deliberately. Previous research shows that mindful eating training helps reduce emotional eating, an important barrier for dietary adherence [18, 19], and evidence suggests that increased mindful eating is associated with decreased fasting glucose levels in participants of a mindful eating weight loss intervention [20]. However, the strategies require extra time and attention from participants, which could be burdensome.

The third strategy we tested was positive affect skills training, which aims to increase the frequency that participants experienced positive emotions. We taught participants skills such as noticing and savoring positive events, gratitude, acts of kindness, positive reappraisal, applying one's personal strengths, and setting attainable goals [21]. To adhere to any dietary intervention, participants need to effectively cope with life stressors, and according to the revised Stress and Coping Theory [22, 23], positive affect can serve as a psychological "time-out" from stress and increase adaptive coping [24-26]. Moreover, interventions that increase the experience of positive affect can reduce depressive symptoms, anxiety, and stress [27], which themselves may decrease treatment adherence [28]. Additionally, hedonic theories of behavior propose that people do more of what they enjoy [29], possibly because positive emotional responses to behaviors increase the motivation and nonconscious desire to engage in those behaviors [30, 31], so participants may be more likely to engage in an intervention that they enjoy. Similarly, previous research has demonstrated an association between higher eating plan satisfaction rates and adherence [32, 33]. However, as with the mindful eating skills, the positive affect skills require extra time and attention from participants, which could be burdensome."

2b) In INTRODUCTION: Specific objectives or hypotheses

Does your paper address CONSORT subitem 2b? *

Copy and paste relevant sections from the manuscript (include quotes in quotation marks "like this" to indicate direct quotes from your manuscript), or elaborate on this item by providing additional information not in the ms, or briefly explain why the item is not applicable/relevant for your study

"The primary aim of this study was to assess whether three supplementary strategies (daily versus monthly dietary self-monitoring, mindful eating, and positive affect skills training) could improve outcomes in this VLC intervention with adults with type 2 diabetes."

METHODS

3a) Description of trial design (such as parallel, factorial) including allocation ratio

Does your paper address CONSORT subitem 3a? *

Copy and paste relevant sections from the manuscript (include quotes in quotation marks "like this" to indicate direct quotes from your manuscript), or elaborate on this item by providing additional information not in the ms, or briefly explain why the item is not applicable/relevant for your study

"This 2x2x2 full factorial experimental design examined the impact of three experimental, 2-level supplementary strategies. Once all baseline measurements had been completed, study staff randomized participants to one of the eight combinations of experimental conditions (Table 1) using a computer program to reveal the next assignment. The order was created using block randomization procedures, with blocks randomly allocated to size 8 or 16 and with the seed numbers of 64655102233242, 64655183677600 from the website Sealed Envelope (Sealed Envelope Ltd. 2015. Create a blocked randomization list. [Online] Available from: https://www.sealedenvelope.com/simple-randomiser/v1/lists [Accessed 14 Jan 2017]). We stratified randomization by gender."

3b) Important changes to methods after trial commencement (such as eligibility criteria), with reasons

Does your paper address CONSORT subitem 3b? *

Copy and paste relevant sections from the manuscript (include quotes in quotation marks "like this" to indicate direct quotes from your manuscript), or elaborate on this item by providing additional information not in the ms, or briefly explain why the item is not applicable/relevant for your study

"Some participants were amid their participation in the trial when the COVID-19 outbreak occurred in the United States (30 of 112 participants or 27%). As the intervention was already completely remote, we were able to continue with the trial, but this may have impacted outcomes."

3b-i) Bug fixes, Downtimes, Content Changes

Bug fixes, Downtimes, Content Changes: ehealth systems are often dynamic systems. A description of changes to methods therefore also includes important changes made on the intervention or comparator during the trial (e.g., major bug fixes or changes in the functionality or content) (5-iii) and other "unexpected events" that may have influenced study design such as staff changes, system failures/downtimes, etc. [2].

subitem not at all important

- 1 ○
- 2 ○
- 3 ◉
- 4 ○
- 5 ○

essential

Clear selection

Does your paper address subitem 3b-i?

Copy and paste relevant sections from the manuscript (include quotes in quotation marks "like this" to indicate direct quotes from your manuscript), or elaborate on this item by providing additional information not in the ms, or briefly explain why the item is not applicable/relevant for your study

This is not relevant. We had none of this.

4a) Eligibility criteria for participants

Does your paper address CONSORT subitem 4a? *

Copy and paste relevant sections from the manuscript (include quotes in quotation marks "like this" to indicate direct quotes from your manuscript), or elaborate on this item by providing additional information not in the ms, or briefly explain why the item is not applicable/relevant for your study

"We invited prospective participants to enroll if they were aged 21 to 70 years, had a baseline HbA1c of 6.5% or higher, had a BMI of 25-45 kg/m2 (based on self-reported height and measured weight from the study-provided scale), had regular access to the internet, were willing to check their email at least once a week, were comfortable reading and writing in English, had no potentially serious comorbidities such as liver or kidney failure, were planning on living in the United States for the duration of the trial, were not vegetarian or vegan, were not on weight-loss medications, and were not taking warfarin or lithium. We also excluded people who were pregnant or breastfeeding, had an untreated thyroid condition, had an untreated mental health condition, had had weight loss surgery in previous year, or were undergoing cancer treatments. Given that this study was conducted remotely, to mitigate the risk of hypoglycemia, we excluded participants who reported taking any anti-glycemic medications other than metformin. Participants who met all entry criteria following the screening process were invited to participate in the trial. "

4a-i) Computer / Internet literacy

Computer / Internet literacy is often an implicit “de facto” eligibility criterion - this should be explicitly clarified.

subitem not at all important

1 ○

2 ○

3 ◉

4 ○

5 ○

essential

Clear selection

Does your paper address subitem 4a-i?

Copy and paste relevant sections from the manuscript (include quotes in quotation marks "like this" to indicate direct quotes from your manuscript), or elaborate on this item by providing additional information not in the ms, or briefly explain why the item is not applicable/relevant for your study

"We invited prospective participants to enroll if they... had regular access to the internet, were willing to check their email at least once a week..."

4a-ii) Open vs. closed, web-based vs. face-to-face assessments:

Open vs. closed, web-based vs. face-to-face assessments: Mention how participants were recruited (online vs. offline), e.g., from an open access website or from a clinic, and clarify if this was a purely web-based trial, or there were face-to-face components (as part of the intervention or for assessment), i.e., to what degree got the study team to know the participant. In online-only trials, clarify if participants were quasi-anonymous and whether having multiple identities was possible or whether technical or logistical measures (e.g., cookies, email confirmation, phone calls) were used to detect/prevent these.

subitem not at all important

1 ○

2 ○

3 ○

4 ◉

5 ○

essential

Clear selection

Does your paper address subitem 4a-ii? *

Copy and paste relevant sections from the manuscript (include quotes in quotation marks "like this" to indicate direct quotes from your manuscript), or elaborate on this item by providing additional information not in the ms, or briefly explain why the item is not applicable/relevant for your study

"We placed advertisements or notices of the research online (including Craigslist, University of Michigan's web-based portal for clinical trials, and ResearchMatch) and sent invitation letters to potentially eligible participants identified from health plan records at Michigan Medicine. Interested prospective participants were directed to the study website, which contained the University of Michigan logo, pertinent study information, and a link to an online self-report screening survey (Qualtrics.com). Those who were eligible for further screening based on their survey responses were asked to provide online electronic consent for the trial and subsequently to complete a second online survey (Qualtrics.com)..."

4a-iii) Information giving during recruitment

Information given during recruitment. Specify how participants were briefed for recruitment and in the informed consent procedures (e.g., publish the informed consent documentation as appendix, see also item X26), as this information may have an effect on user self-selection, user expectation and may also bias results.

subitem not at all important

1 ○

2 ○

3 ○

4 ◉

5 ○

essential

Clear selection

Does your paper address subitem 4a-iii?

Copy and paste relevant sections from the manuscript (include quotes in quotation marks "like this" to indicate direct quotes from your manuscript), or elaborate on this item by providing additional information not in the ms, or briefly explain why the item is not applicable/relevant for your study

"Those who were eligible for further screening based on their survey responses were asked to provide online electronic consent for the trial..."

4b) Settings and locations where the data were collected

Does your paper address CONSORT subitem 4b? *

Copy and paste relevant sections from the manuscript (include quotes in quotation marks "like this" to indicate direct quotes from your manuscript), or elaborate on this item by providing additional information not in the ms, or briefly explain why the item is not applicable/relevant for your study

"...online self-report screening survey (Qualtrics.com)... a fingerstick self-collected mail-in blood test kit for glycated hemoglobin (HbA1c) test from DTI Laboratories, Inc; and 3 days of dietary self-monitoring on MyFitnessPal. We also mailed participants a body weight scale that connects to its own cellular network (BodyTrace)."

4b-i) Report if outcomes were (self-)assessed through online questionnaires

Clearly report if outcomes were (self-)assessed through online questionnaires (as common in web-based trials) or otherwise.

subitem not at all important

1 ○

2 ○

3 ○

4 ◉

5 ○

essential

Clear selection

Does your paper address subitem 4b-i? *

Copy and paste relevant sections from the manuscript (include quotes in quotation marks "like this" to indicate direct quotes from your manuscript), or elaborate on this item by providing additional information not in the ms, or briefly explain why the item is not applicable/relevant for your study

"Glycemic Control
We measured our primary outcome, change in glycemic control, with an at-home HbA1c kit from DTI Laboratories, Inc.
Weight
We assessed one of our secondary outcomes, change in percent body weight, using the scale we had mailed to participants (we asked participants to stand on the scale twice in five minutes at each time period, and we used the average of these two measurements as their recorded weight).
Depressive Symptoms
We assessed one of our secondary outcomes, change in depressive symptoms, using the PHQ-8 [34].
Classes Viewed
We tracked the total number of classes that were viewed by participants.
Dietary Adherence
With each class, we asked whether, based on self-assessment, participants were following a VLC diet rated from 1= “not at all” to 7= “very much so” and used the average of these ratings as an indicator of self-reported dietary adherence.
Intervention Satisfaction
At month 12, we asked participants, “How would you rate your overall satisfaction with the program?” (Response options ranged from 1= “not at all satisfied” to 7= “very satisfied”).
Metformin Use
We considered a participant to have changed their metformin dose at 12 months if they mentioned the medications change in their surveys, using the most recent description of the changes as their outcome in the trial.
Qualitative Feedback
We asked participants, using open-ended survey questions, about their thoughts about the intervention itself, their health changes, and the responses they had previously received from their physicians."

4b-ii) Report how institutional affiliations are displayed

Report how institutional affiliations are displayed to potential participants [on ehealth media], as affiliations with prestigious hospitals or universities may affect volunteer rates, use, and reactions with regards to an intervention.(Not a required item – describe only if this may bias results)

subitem not at all important

1 ○

2 ○

3 ◉

4 ○

5 ○

essential

Clear selection

Does your paper address subitem 4b-ii?

Copy and paste relevant sections from the manuscript (include quotes in quotation marks "like this" to indicate direct quotes from your manuscript), or elaborate on this item by providing additional information not in the ms, or briefly explain why the item is not applicable/relevant for your study

"Interested prospective participants were directed to the study website, which contained the University of Michigan logo, pertinent study information, and a link to an online self-report screening survey (Qualtrics.com)."

5) The interventions for each group with sufficient details to allow replication, including how and when they were actually administered

5-i) Mention names, credential, affiliations of the developers, sponsors, and owners

Mention names, credential, affiliations of the developers, sponsors, and owners [6] (if authors/evaluators are owners or developer of the software, this needs to be declared in a “Conflict of interest” section or mentioned elsewhere in the manuscript).

subitem not at all important

1 ○

2 ○

3 ◉

4 ○

5 ○

essential

Clear selection

Does your paper address subitem 5-i?

Copy and paste relevant sections from the manuscript (include quotes in quotation marks "like this" to indicate direct quotes from your manuscript), or elaborate on this item by providing additional information not in the ms, or briefly explain why the item is not applicable/relevant for your study

"LS’s partner, HB, is an inventor of software used in this study, which purchased a software services agreement for its use."

5-ii) Describe the history/development process

Describe the history/development process of the application and previous formative evaluations (e.g., focus groups, usability testing), as these will have an impact on adoption/use rates and help with interpreting results.

subitem not at all important

1 ○

2 ○

3 ●

4 ○

5 ○

essential

Clear selection

Does your paper address subitem 5-ii?

Copy and paste relevant sections from the manuscript (include quotes in quotation marks "like this" to indicate direct quotes from your manuscript), or elaborate on this item by providing additional information not in the ms, or briefly explain why the item is not applicable/relevant for your study

We reference our previous, similar research: Saslow, L.R., et al., Intervention Enhancement Strategies Among Adults With Type 2 Diabetes in a Very Low-Carbohydrate Web-Based Program: Evaluating the Impact With a Randomized Trial. JMIR Diabetes, 2020. 5(3): p. e15835.

5-iii) Revisions and updating

Revisions and updating. Clearly mention the date and/or version number of the application/intervention (and comparator, if applicable) evaluated, or describe whether the intervention underwent major changes during the evaluation process, or whether the development and/or content was “frozen” during the trial. Describe dynamic components such as news feeds or changing content which may have an impact on the replicability of the intervention (for unexpected events see item 3b).

subitem not at all important

1 ○

2 ○

3 ○

4 ◉

5 ○

essential

Clear selection

Does your paper address subitem 5-iii?

Copy and paste relevant sections from the manuscript (include quotes in quotation marks "like this" to indicate direct quotes from your manuscript), or elaborate on this item by providing additional information not in the ms, or briefly explain why the item is not applicable/relevant for your study

Not applicable. The intervention did not change.

5-iv) Quality assurance methods

Provide information on quality assurance methods to ensure accuracy and quality of information provided [1], if applicable.

subitem not at all important

1 ○

2 ○

3 ◉

4 ○

5 ○

essential

Clear selection

Does your paper address subitem 5-iv?

Copy and paste relevant sections from the manuscript (include quotes in quotation marks "like this" to indicate direct quotes from your manuscript), or elaborate on this item by providing additional information not in the ms, or briefly explain why the item is not applicable/relevant for your study

"Participants also had email access to a dietary coach (either author K.R. or M. P.), as coaches have generally been found to be effective additions to behavioral interventions [35]. Both coaches had extensive experience with a VLC diet, and all messages were checked for fidelity by the first author, L.S., before being sent."

5-v) Ensure replicability by publishing the source code, and/or providing screenshots/screen-capture video, and/or providing flowcharts of the algorithms used

Ensure replicability by publishing the source code, and/or providing screenshots/screen-capture video, and/or providing flowcharts of the algorithms used. Replicability (i.e., other researchers should in principle be able to replicate the study) is a hallmark of scientific reporting.

subitem not at all important

1 ○

2 ○

3 ◉

4 ○

5 ○

essential

Clear selection

Does your paper address subitem 5-v?

Copy and paste relevant sections from the manuscript (include quotes in quotation marks "like this" to indicate direct quotes from your manuscript), or elaborate on this item by providing additional information not in the ms, or briefly explain why the item is not applicable/relevant for your study

We describe the complicated, 12-month intervention.

5-vi) Digital preservation

Digital preservation: Provide the URL of the application, but as the intervention is likely to change or disappear over the course of the years; also make sure the intervention is archived (Internet Archive, webcitation.org, and/or publishing the source code or screenshots/videos alongside the article). As pages behind login screens cannot be archived, consider creating demo pages which are accessible without login.

subitem not at all important

1 ○

2 ○

3 ◉

4 ○

5 ○

essential

Clear selection

Does your paper address subitem 5-vi?

Copy and paste relevant sections from the manuscript (include quotes in quotation marks "like this" to indicate direct quotes from your manuscript), or elaborate on this item by providing additional information not in the ms, or briefly explain why the item is not applicable/relevant for your study

We did not preserve the intervention digitally.

5-vii) Access

Access: Describe how participants accessed the application, in what setting/context, if they had to pay (or were paid) or not, whether they had to be a member of specific group. If known, describe how participants obtained "access to the platform and Internet" [1]. To ensure access for editors/reviewers/readers, consider to provide a "backdoor" login account or demo mode for reviewers/readers to explore the application (also important for archiving purposes, see vi).

subitem not at all important

1 ○

2 ○

3 ◉

4 ○

5 ○

essential

Clear selection

Does your paper address subitem 5-vii? *

Copy and paste relevant sections from the manuscript (include quotes in quotation marks "like this" to indicate direct quotes from your manuscript), or elaborate on this item by providing additional information not in the ms, or briefly explain why the item is not applicable/relevant for your study

"We measured outcomes at baseline and at 4, 8, and 12 months after baseline. As an incentive for continued participation, we paid participants US $25 for completing their outcome measurements at 4 months, US $25 at 8 months, and US $50 at 12 months." "Once participants were assigned to the different intervention strategies, we e-mailed all participants links to the core online VLC intervention materials throughout the 12-month intervention, weekly for the first 4 months and then every 2 weeks for the remaining 8 months, for a total of 32 emails. Each of the 32 sets of materials focused on a different topic related to following a VLC diet. Emailed links connected participants to a) a short survey to assess intervention-related dietary adherence and any health concerns, b) a short, embedded video teaching class topics (such as managing their diet during holidays or shifting particular meals to be VLC, etc.), c) downloadable handouts to accompany the video, and d) links to external online resources supporting class topics. Transcripts of the embedded videos were also provided."

5-viii) Mode of delivery, features/functionalities/components of the intervention and comparator, and the theoretical framework

Describe mode of delivery, features/functionalities/components of the intervention and comparator, and the theoretical framework [6] used to design them (instructional strategy [1], behaviour change techniques, persuasive features, etc., see e.g., [7, 8] for terminology). This includes an in-depth description of the content (including where it is coming from and who developed it) [1]," whether [and how] it is tailored to individual circumstances and allows users to track their progress and receive feedback" [6]. This also includes a description of communication delivery channels and – if computer-mediated communication is a component – whether communication was synchronous or asynchronous [6]. It also includes information on presentation strategies [1], including page design principles, average amount of text on pages, presence of hyperlinks to other resources, etc. [1].

subitem not at all important

- 1 ○
- 2 ○
- 3 ◉
- 4 ○
- 5 ○

essential

Clear selection

Does your paper address subitem 5-viii? *

Copy and paste relevant sections from the manuscript (include quotes in quotation marks "like this" to indicate direct quotes from your manuscript), or elaborate on this item by providing additional information not in the ms, or briefly explain why the item is not applicable/relevant for your study

"Once participants were assigned to the different intervention strategies, we e-mailed all participants links to the core online VLC intervention materials throughout the 12-month intervention, weekly for the first 4 months and then every 2 weeks for the remaining 8 months, for a total of 32 emails. Each of the 32 sets of materials focused on a different topic related to following a VLC diet. Emailed links connected participants to a) a short survey to assess intervention-related dietary adherence and any health concerns, b) a short, embedded video teaching class topics (such as managing their diet during holidays or shifting particular meals to be VLC, etc.), c) downloadable handouts to accompany the video, and d) links to external online resources supporting class topics. Transcripts of the embedded videos were also provided." "Dietary Self-Monitoring
We assigned all participants to self-monitor their diet using the free web-based and/or mobile application MyFitnessPal [42], which has a wide variety of foods in its database that are common to the diet assigned in this trial (reducing participant burden and increasing accuracy), at varying frequencies. Participants were randomized to track their diet daily or for monthly (which we defined as 3 days every 4 weeks).
Mindful Eating Skills
We assigned half of participants to receive training in mindful eating, how to practice the skills in everyday life, research supporting the use of the skills, how and why the skills were expected to help, and targeted suggestions for practicing the skills. We asked participants to focus on consciously savoring their food, eating more slowly, and noticing the textures, flavors, and aromas of their food more carefully. For example, during one session, we asked them to practice slowly savoring their food with a snack and encouraged them to practice this skill during at least one meal a day over the following week.
Positive Affect Skills
We assigned half of participants to receive training in positive affect skills. They were taught how to practice them in everyday life, research supporting the use of the skills, how and why the skills were expected to help, and targeted suggestions for practicing the skills. The skills we taught included noticing and savoring positive events, gratitude, positive reappraisal, and setting attainable goals, similar to our previous research [21]."

5-ix) Describe use parameters

Describe use parameters (e.g., intended “doses” and optimal timing for use). Clarify what instructions or recommendations were given to the user, e.g., regarding timing, frequency, heaviness of use, if any, or was the intervention used ad libitum.

subitem not at all important

1 ○

2 ○

3 ◉

4 ○

5 ○

essential

Clear selection

Does your paper address subitem 5-ix?

Copy and paste relevant sections from the manuscript (include quotes in quotation marks "like this" to indicate direct quotes from your manuscript), or elaborate on this item by providing additional information not in the ms, or briefly explain why the item is not applicable/relevant for your study

"Once participants were assigned to the different intervention strategies, we e-mailed all participants links to the core online VLC intervention materials throughout the 12-month intervention, weekly for the first 4 months and then every 2 weeks for the remaining 8 months, for a total of 32 emails."

5-x) Clarify the level of human involvement

Clarify the level of human involvement (care providers or health professionals, also technical assistance) in the e-intervention or as co-intervention (detail number and expertise of professionals involved, if any, as well as “type of assistance offered, the timing and frequency of the support, how it is initiated, and the medium by which the assistance is delivered”. It may be necessary to distinguish between the level of human involvement required for the trial, and the level of human involvement required for a routine application outside of a RCT setting (discuss under item 21 – generalizability).

subitem not at all important

1 ○

2 ○

3 ◉

4 ○

5 ○

essential

Clear selection

Does your paper address subitem 5-x?

Copy and paste relevant sections from the manuscript (include quotes in quotation marks "like this" to indicate direct quotes from your manuscript), or elaborate on this item by providing additional information not in the ms, or briefly explain why the item is not applicable/relevant for your study

"Participants also had email access to a dietary coach (either author K.R. or M. P.), as coaches have generally been found to be effective additions to behavioral interventions [35]. Both coaches had extensive experience with a VLC diet, and all messages were checked for fidelity by the first author, L.S., before being sent. Whenever the participants emailed the coaches questions, participants would receive prompt replies with support and resources. Overall, the coaches emailed participants a minimum of every two weeks. Participants also received a body weight scale at the start of their participation, and we asked participants to monitor their body weight regularly, aiming for weighing themselves at least weekly. Coaches used this information to monitor participant success and tailor support."

5-xi) Report any prompts/reminders used

Report any prompts/reminders used: Clarify if there were prompts (letters, emails, phone calls, SMS) to use the application, what triggered them, frequency etc. It may be necessary to distinguish between the level of prompts/reminders required for the trial, and the level of prompts/reminders for a routine application outside of a RCT setting (discuss under item 21 – generalizability).

subitem not at all important

1 ○

2 ○

3 ◉

4 ○

5 ○

essential

Clear selection

Does your paper address subitem 5-xi? *

Copy and paste relevant sections from the manuscript (include quotes in quotation marks "like this" to indicate direct quotes from your manuscript), or elaborate on this item by providing additional information not in the ms, or briefly explain why the item is not applicable/relevant for your study

"To encourage the adoption and maintenance of the new intervention-related behaviors, we sent participants text messages up to 5 times a week about the targeted behaviors and skills, depending on which supplemental strategies they were randomized to, as reminders about targeted behaviors are tied to greater behavioral adherence [37]. To help participants change their dietary patterns, we mailed cookbooks to participants: Keto Living 3 Cookbook: Lose Weight with 101 All New Delicious and Low Carb Ketogenic Recipes [38] at baseline, Bacon & Butter, the Ultimate Ketogenic Diet Cookbook [39] at month 3, The Wicked Good Ketogenic Diet Cookbook: Easy, Whole Food Keto Recipes for Any Budget [40] at month 6, and The Everyday Ketogenic Kitchen With More Than 150 Inspirational Low-Carb, High-Fat Recipes to Maximize Your Health [41] at month 10."

5-xii) Describe any co-interventions (incl. training/support)

Describe any co-interventions (incl. training/support): Clearly state any interventions that are provided in addition to the targeted eHealth intervention, as ehealth intervention may not be designed as stand-alone intervention. This includes training sessions and support [1]. It may be necessary to distinguish between the level of training required for the trial, and the level of training for a routine application outside of a RCT setting (discuss under item 21 – generalizability.

subitem not at all important

1 ○

2 ○

3 ○

4 ◉

5 ○

essential

Clear selection

Does your paper address subitem 5-xii? *

Copy and paste relevant sections from the manuscript (include quotes in quotation marks "like this" to indicate direct quotes from your manuscript), or elaborate on this item by providing additional information not in the ms, or briefly explain why the item is not applicable/relevant for your study

"Dietary Self-Monitoring
We assigned all participants to self-monitor their diet using the free web-based and/or mobile application MyFitnessPal [42], which has a wide variety of foods in its database that are common to the diet assigned in this trial (reducing participant burden and increasing accuracy), at varying frequencies. Participants were randomized to track their diet daily or for monthly (which we defined as 3 days every 4 weeks).
Mindful Eating Skills
We assigned half of participants to receive training in mindful eating, how to practice the skills in everyday life, research supporting the use of the skills, how and why the skills were expected to help, and targeted suggestions for practicing the skills. We asked participants to focus on consciously savoring their food, eating more slowly, and noticing the textures, flavors, and aromas of their food more carefully. For example, during one session, we asked them to practice slowly savoring their food with a snack and encouraged them to practice this skill during at least one meal a day over the following week.
Positive Affect Skills
We assigned half of participants to receive training in positive affect skills. They were taught how to practice them in everyday life, research supporting the use of the skills, how and why the skills were expected to help, and targeted suggestions for practicing the skills. The skills we taught included noticing and savoring positive events, gratitude, positive reappraisal, and setting attainable goals, similar to our previous research [21]."

6a) Completely defined pre-specified primary and secondary outcome measures, including how and when they were assessed

Does your paper address CONSORT subitem 6a? *

Copy and paste relevant sections from the manuscript (include quotes in quotation marks "like this" to indicate direct quotes from your manuscript), or elaborate on this item by providing additional information not in the ms, or briefly explain why the item is not applicable/relevant for your study

"Glycemic Control
We measured our primary outcome, change in glycemic control at 12 months from baseline, with an at-home HbA1c kit from DTI Laboratories, Inc.
Weight
We assessed one of our secondary outcomes, change in percent body weight at 12 months from baseline, using the scale we had mailed to participants (we asked participants to stand on the scale twice in five minutes at each time period, and we used the average of these two measurements as their recorded weight).
Depressive Symptoms
We assessed one of our secondary outcomes, change in depressive symptoms at 12 months from baseline, using the PHQ-8 [34]."

6a-i) Online questionnaires: describe if they were validated for online use and apply CHERRIES items to describe how the questionnaires were designed/deployed

If outcomes were obtained through online questionnaires, describe if they were validated for online use and apply CHERRIES items to describe how the questionnaires were designed/deployed [9].

subitem not at all important

1 ○

2 ○

3 ◉

4 ○

5 ○

essential

Clear selection

Does your paper address subitem 6a-i?

Copy and paste relevant sections from manuscript text

"Depressive Symptoms
We assessed one of our secondary outcomes, change in depressive symptoms at 12 months from baseline, using the PHQ-8 [34].
Dietary Adherence
With each class, we asked whether, based on self-assessment, participants were following a VLC diet rated from 1= "not at all" to 7= "very much so" and used the average of these ratings as an indicator of self-reported dietary adherence.
Intervention Satisfaction
At month 12, we asked participants, "How would you rate your overall satisfaction with the program?" (Response options ranged from 1= "not at all satisfied" to 7= "very satisfied")."

6a-ii) Describe whether and how "use" (including intensity of use/dosage) was defined/measured/monitored

Describe whether and how "use" (including intensity of use/dosage) was defined/measured/monitored (logins, logfile analysis, etc.). Use/adoption metrics are important process outcomes that should be reported in any ehealth trial.

subitem not at all important

1 ○

2 ○

3 ◉

4 ○

5 ○

essential

Clear selection

Does your paper address subitem 6a-ii?

Copy and paste relevant sections from manuscript text

"Those who were eligible for further screening based on their survey responses were asked to provide online electronic consent for the trial and subsequently to complete a second online survey (Qualtrics.com) which included the eight-item Patient Health Questionnaire (PHQ-8) to measure depressive symptoms [34]; a fingerstick self-collected mail-in blood test kit for glycated hemoglobin (HbA1c) test from DTI Laboratories, Inc; and 3 days of dietary self-monitoring on MyFitnessPal. We also mailed participants a body weight scale that connects to its own cellular network (BodyTrace). "

6a-iii) Describe whether, how, and when qualitative feedback from participants was obtained

Describe whether, how, and when qualitative feedback from participants was obtained (e.g., through emails, feedback forms, interviews, focus groups).

subitem not at all important

1 ○

2 ○

3 ●

4 ○

5 ○

essential

Clear selection

Does your paper address subitem 6a-iii?

Copy and paste relevant sections from manuscript text

"We asked participants, using open-ended survey questions, about their thoughts about the intervention itself, their health changes, and the responses they had previously received from their physicians."

6b) Any changes to trial outcomes after the trial commenced, with reasons

Does your paper address CONSORT subitem 6b? *

Copy and paste relevant sections from the manuscript (include quotes in quotation marks "like this" to indicate direct quotes from your manuscript), or elaborate on this item by providing additional information not in the ms, or briefly explain why the item is not applicable/relevant for your study

We did not have changes to trial outcomes after the trial commenced.

7a) How sample size was determined

NPT: When applicable, details of whether and how the clustering by care provides or centers was addressed

7a-i) Describe whether and how expected attrition was taken into account when calculating the sample size

Describe whether and how expected attrition was taken into account when calculating the sample size.

subitem not at all important

1 ○

2 ○

3 ◉

4 ○

5 ○

essential

Clear selection

Does your paper address subitem 7a-i?

Copy and paste relevant sections from manuscript title (include quotes in quotation marks "like this" to indicate direct quotes from your manuscript), or elaborate on this item by providing additional information not in the ms, or briefly explain why the item is not applicable/relevant for your study

"Sample size calculations were performed using a SAS macro written by Dr. Linda Collins and her team, designed for factorial trials [43]. Our goal was to screen for supplemental strategies that had at least a medium effect size (a Cohen's d of 0.5). The sample size was also estimated using a desired power of 0.8, a two-tailed p value of 0.05, and a pretest-posttest correlation of HbA1c (the primary outcome of interest) of between 0.30 and 0.40 (based on pilot data). This led to a sample size goal of 108-117 of analyzable cases, which, with a planned attrition rate of 20%, would be 130-140 randomized participants. Our final sample size of 112 was therefore slightly underpowered, after attrition, to detect changes between strategies based on our sample size estimates."

7b) When applicable, explanation of any interim analyses and stopping guidelines

Does your paper address CONSORT subitem 7b? *

Copy and paste relevant sections from the manuscript (include quotes in quotation marks "like this" to indicate direct quotes from your manuscript), or elaborate on this item by providing additional information not in the ms, or briefly explain why the item is not applicable/relevant for your study

We did not have any interim analyses and stopping guidelines.

8a) Method used to generate the random allocation sequence

NPT: When applicable, how care providers were allocated to each trial group

Does your paper address CONSORT subitem 8a? *

Copy and paste relevant sections from the manuscript (include quotes in quotation marks "like this" to indicate direct quotes from your manuscript), or elaborate on this item by providing additional information not in the ms, or briefly explain why the item is not applicable/relevant for your study

"The order was created using block randomization procedures, with blocks randomly allocated to size 8 or 16 and with the seed numbers of 64655102233242, 64655183677600 from the website Sealed Envelope (Sealed Envelope Ltd. 2015. Create a blocked randomization list. [Online] Available from: https://www.sealedenvelope.com/simple-randomiser/v1/lists [Accessed 14 Jan 2017])."

8b) Type of randomisation; details of any restriction (such as blocking and block size)

Does your paper address CONSORT subitem 8b? *

Copy and paste relevant sections from the manuscript (include quotes in quotation marks "like this" to indicate direct quotes from your manuscript), or elaborate on this item by providing additional information not in the ms, or briefly explain why the item is not applicable/relevant for your study

"Once all baseline measurements had been completed, study staff randomized participants to one of the eight combinations of experimental conditions (Table 1) using a computer program to reveal the next assignment. The order was created using block randomization procedures, with blocks randomly allocated to size 8 or 16..."

9) Mechanism used to implement the random allocation sequence (such as sequentially numbered containers), describing any steps taken to conceal the sequence until interventions were assigned

Does your paper address CONSORT subitem 9? *

Copy and paste relevant sections from the manuscript (include quotes in quotation marks "like this" to indicate direct quotes from your manuscript), or elaborate on this item by providing additional information not in the ms, or briefly explain why the item is not applicable/relevant for your study

"Once all baseline measurements had been completed, study staff randomized participants to one of the eight combinations of experimental conditions (Table 1) using a computer program to reveal the next assignment."

10) Who generated the random allocation sequence, who enrolled participants, and who assigned participants to interventions

Does your paper address CONSORT subitem 10? *

Copy and paste relevant sections from the manuscript (include quotes in quotation marks "like this" to indicate direct quotes from your manuscript), or elaborate on this item by providing additional information not in the ms, or briefly explain why the item is not applicable/relevant for your study

"The order was created using block randomization procedures, with blocks randomly allocated to size 8 or 16 and with the seed numbers of 64655102233242, 64655183677600 from the website Sealed Envelope (Sealed Envelope Ltd. 2015....)"

11a) If done, who was blinded after assignment to interventions (for example, participants, care providers, those assessing outcomes) and how

NPT: Whether or not administering co-interventions were blinded to group assignment

11a-i) Specify who was blinded, and who wasn't

Specify who was blinded, and who wasn't. Usually, in web-based trials it is not possible to blind the participants [1, 3] (this should be clearly acknowledged), but it may be possible to blind outcome assessors, those doing data analysis or those administering co-interventions (if any).

subitem not at all important

1 ○

2 ○

3 ◉

4 ○

5 ○

essential

Clear selection

Does your paper address subitem 11a-i? *

Copy and paste relevant sections from the manuscript (include quotes in quotation marks "like this" to indicate direct quotes from your manuscript), or elaborate on this item by providing additional information not in the ms, or briefly explain why the item is not applicable/relevant for your study

"Glycemic Control
We measured our primary outcome, change in glycemic control at 12 months from baseline, with an at-home HbA1c kit (DTI Laboratories, Inc, Thomasville, GA). This company was masked to the intervention design.
Weight
We assessed one of our secondary outcomes, change in percent body weight at 12 months from baseline, using the scale we had mailed to participants (BodyTrace, Palo Alto, CA). This company was masked to the intervention design. We asked participants to stand on the scale twice in five minutes at each time period, and we used the average of these two measurements as their recorded weight).
Depressive Symptoms
We assessed one of our secondary outcomes, change in depressive symptoms at 12 months from baseline, using the PHQ-8 [34]. This was part of a web-based survey (Qualtrics.com, Provo, UT). This company was masked to the intervention design.
Classes Viewed
We tracked the total number of classes that were viewed by participants.
Dietary Adherence
With each class, we asked whether, based on self-assessment, participants were following a VLC diet rated from 1= “not at all” to 7= “very much so” and used the average of these ratings as an indicator of self-reported dietary adherence.
Intervention Satisfaction
At month 12, we asked participants, “How would you rate your overall satisfaction with the program?” (Response options ranged from 1= “not at all satisfied” to 7= “very satisfied”). This was part of a web-based survey (Qualtrics.com, Provo, UT). "

11a-ii) Discuss e.g., whether participants knew which intervention was the "intervention of interest" and which one was the "comparator"

Informed consent procedures (4a-ii) can create biases and certain expectations - discuss e.g., whether participants knew which intervention was the "intervention of interest" and which one was the "comparator".

subitem not at all important

1 ○

2 ○

3 ◉

4 ○

5 ○

essential

Clear selection

Does your paper address subitem 11a-ii?

Copy and paste relevant sections from the manuscript (include quotes in quotation marks "like this" to indicate direct quotes from your manuscript), or elaborate on this item by providing additional information not in the ms, or briefly explain why the item is not applicable/relevant for your study

"Participants were aware of the study design, but we did not explicitly describe that participants were in the on or off group of each behavioral strategy."

11b) If relevant, description of the similarity of interventions

(this item is usually not relevant for ehealth trials as it refers to similarity of a placebo or sham intervention to a active medication/intervention)

Does your paper address CONSORT subitem 11b? *

Copy and paste relevant sections from the manuscript (include quotes in quotation marks "like this" to indicate direct quotes from your manuscript), or elaborate on this item by providing additional information not in the ms, or briefly explain why the item is not applicable/relevant for your study

"We randomized participants to receive a VLC diet and one of eight possible combinations of the three supplemental strategies: dietary self-monitoring, mindful eating, or positive affect skills. The program materials were modified for each supplemental strategy, including different content added to the videos, handouts, and text messages."

12a) Statistical methods used to compare groups for primary and secondary outcomes

NPT: When applicable, details of whether and how the clustering by care providers or centers was addressed

Does your paper address CONSORT subitem 12a? *

Copy and paste relevant sections from the manuscript (include quotes in quotation marks "like this" to indicate direct quotes from your manuscript), or elaborate on this item by providing additional information not in the ms, or briefly explain why the item is not applicable/relevant for your study

"To assess 12-month intervention changes, we conducted complete-case analyses, excluding participants who did not complete the relevant 12-month assessment. Collapsing across all participants, we examined pre-post changes in HbA1c (our primary outcome), percent weight, PHQ-8, as well as the number of classes participants viewed, self-reported dietary adherence, and intervention satisfaction, using between subjects t-tests using SPSS 28.0 (IBM Corp., Armonk, N.Y., USA). We explored outcomes using intent-to-treat analyses (n=112) with linear mixed regression models using SPSS 28.0. This approach makes use of all available data at all timepoints, with the outcomes as dependent variables, time (pre, post), and all three strategies in a full-factorial design. We explored overall changes across strategies using within subjects t-tests using SPSS. For the qualitative results, we examined open-ended comments and summarized common themes and exemplar quotes."

12a-i) Imputation techniques to deal with attrition / missing values

Imputation techniques to deal with attrition / missing values: Not all participants will use the intervention/comparator as intended and attrition is typically high in ehealth trials. Specify how participants who did not use the application or dropped out from the trial were treated in the statistical analysis (a complete case analysis is strongly discouraged, and simple imputation techniques such as LOCF may also be problematic [4]).

subitem not at all important

1 ○

2 ○

3 ○

4 ◉

5 ○

essential

Clear selection

Does your paper address subitem 12a-i? *

Copy and paste relevant sections from the manuscript (include quotes in quotation marks "like this" to indicate direct quotes from your manuscript), or elaborate on this item by providing additional information not in the ms, or briefly explain why the item is not applicable/relevant for your study

"To assess 12-month intervention changes, we conducted complete-case analyses, excluding participants who did not complete the relevant 12-month assessment. Collapsing across all participants, we examined pre-post changes in HbA1c (our primary outcome), percent weight, PHQ-8, as well as the number of classes participants viewed, self-reported dietary adherence, and intervention satisfaction, using between subjects t-tests using SPSS 28.0 (IBM Corp., Armonk, N.Y., USA). We explored outcomes using intent-to-treat analyses (n=112) with linear mixed regression models using SPSS 28.0. This approach makes use of all available data at all timepoints, with the outcomes as dependent variables, time (pre, post), and all three strategies in a full-factorial design."

12b) Methods for additional analyses, such as subgroup analyses and adjusted analyses

Does your paper address CONSORT subitem 12b? *

Copy and paste relevant sections from the manuscript (include quotes in quotation marks "like this" to indicate direct quotes from your manuscript), or elaborate on this item by providing additional information not in the ms, or briefly explain why the item is not applicable/relevant for your study

We did not do these other analyses.

X26) REB/IRB Approval and Ethical Considerations [recommended as subheading under "Methods"] (not a CONSORT item)

X26-i) Comment on ethics committee approval

subitem not at all important

1 ○

2 ○

3 ○

4 ◉

5 ○

essential

Clear selection

Does your paper address subitem X26-i?

Copy and paste relevant sections from the manuscript (include quotes in quotation marks "like this" to indicate direct quotes from your manuscript), or elaborate on this item by providing additional information not in the ms, or briefly explain why the item is not applicable/relevant for your study

"The institutional review board at the University of Michigan approved this research (HUM00115537)."

x26-ii) Outline informed consent procedures

Outline informed consent procedures e.g., if consent was obtained offline or online (how? Checkbox, etc.?), and what information was provided (see 4a-ii). See [6] for some items to be included in informed consent documents.

subitem not at all important

1 ○

2 ○

3 ○

4 ◉

5 ○

essential

Clear selection

Does your paper address subitem X26-ii?

Copy and paste relevant sections from the manuscript (include quotes in quotation marks "like this" to indicate direct quotes from your manuscript), or elaborate on this item by providing additional information not in the ms, or briefly explain why the item is not applicable/relevant for your study

"They were consented using an approved web-based consent form that described the study procedures and goals."

X26-iii) Safety and security procedures

Safety and security procedures, incl. privacy considerations, and any steps taken to reduce the likelihood or detection of harm (e.g., education and training, availability of a hotline)

subitem not at all important

1 ○

2 ○

3 ◉

4 ○

5 ○

essential

Clear selection

Does your paper address subitem X26-iii?

Copy and paste relevant sections from the manuscript (include quotes in quotation marks "like this" to indicate direct quotes from your manuscript), or elaborate on this item by providing additional information not in the ms, or briefly explain why the item is not applicable/relevant for your study

"Given that this study was conducted remotely, to mitigate the risk of hypoglycemia, we excluded participants who reported taking any anti-glycemic medications other than metformin."

RESULTS

13a) For each group, the numbers of participants who were randomly assigned, received intended treatment, and were analysed for the primary outcome

NPT: The number of care providers or centers performing the intervention in each group and the number of patients treated by each care provider in each center

Does your paper address CONSORT subitem 13a? *

Copy and paste relevant sections from the manuscript (include quotes in quotation marks "like this" to indicate direct quotes from your manuscript), or elaborate on this item by providing additional information not in the ms, or briefly explain why the item is not applicable/relevant for your study

This is in a CONSORT flow diagram.

13b) For each group, losses and exclusions after randomisation, together with reasons

Does your paper address CONSORT subitem 13b? (NOTE: Preferably, this is shown in a CONSORT flow diagram) *

Copy and paste relevant sections from the manuscript (include quotes in quotation marks "like this" to indicate direct quotes from your manuscript), or elaborate on this item by providing additional information not in the ms, or briefly explain why the item is not applicable/relevant for your study

This is in a CONSORT flow diagram.

13b-i) Attrition diagram

Strongly recommended: An attrition diagram (e.g., proportion of participants still logging in or using the intervention/comparator in each group plotted over time, similar to a survival curve) or other figures or tables demonstrating usage/dose/engagement.

subitem not at all important

- 1 ○
- 2 ○
- 3 ●
- 4 ○
- 5 ○

essential

Clear selection

Does your paper address subitem 13b-i?

Copy and paste relevant sections from the manuscript or cite the figure number if applicable (include quotes in quotation marks "like this" to indicate direct quotes from your manuscript), or elaborate on this item by providing additional information not in the ms, or briefly explain why the item is not applicable/relevant for your study

This is in a CONSORT flow diagram.

14a) Dates defining the periods of recruitment and follow-up

Does your paper address CONSORT subitem 14a? *

Copy and paste relevant sections from the manuscript (include quotes in quotation marks "like this" to indicate direct quotes from your manuscript), or elaborate on this item by providing additional information not in the ms, or briefly explain why the item is not applicable/relevant for your study

"We recruited participants between 2/4/17 and 2/28/20, and completed data collection by 6/4/21."

14a-i) Indicate if critical “secular events” fell into the study period

Indicate if critical “secular events” fell into the study period, e.g., significant changes in Internet resources available or “changes in computer hardware or Internet delivery resources”

subitem not at all important

- 1 ○
- 2 ○
- 3 ○
- 4 ◉
- 5 ○

essential

Clear selection

Does your paper address subitem 14a-i?

Copy and paste relevant sections from the manuscript (include quotes in quotation marks "like this" to indicate direct quotes from your manuscript), or elaborate on this item by providing additional information not in the ms, or briefly explain why the item is not applicable/relevant for your study

"Another limitation of the trial was that 27% of the participants were in the trial when Covid-19 began in the United States in 2020, so our conclusions about which supplementary strategies might be most helpful may have been influenced by this."

14b) Why the trial ended or was stopped (early)

Does your paper address CONSORT subitem 14b? *

Copy and paste relevant sections from the manuscript (include quotes in quotation marks "like this" to indicate direct quotes from your manuscript), or elaborate on this item by providing additional information not in the ms, or briefly explain why the item is not applicable/relevant for your study

"In addition, in part due to Covid-19, we did not recruit our intended number of participants, so the trial was underpowered."

15) A table showing baseline demographic and clinical characteristics for each group

NPT: When applicable, a description of care providers (case volume, qualification, expertise, etc.) and centers (volume) in each group

Does your paper address CONSORT subitem 15? *

Copy and paste relevant sections from the manuscript (include quotes in quotation marks "like this" to indicate direct quotes from your manuscript), or elaborate on this item by providing additional information not in the ms, or briefly explain why the item is not applicable/relevant for your study

We show this information in Table 2.

15-i) Report demographics associated with digital divide issues

In ehealth trials it is particularly important to report demographics associated with digital divide issues, such as age, education, gender, social-economic status, computer/Internet/ehealth literacy of the participants, if known.

subitem not at all important

1 ○

2 ○

3 ○

4 ◉

5 ○

essential

Clear selection

Does your paper address subitem 15-i? *

Copy and paste relevant sections from the manuscript (include quotes in quotation marks "like this" to indicate direct quotes from your manuscript), or elaborate on this item by providing additional information not in the ms, or briefly explain why the item is not applicable/relevant for your study

We report on age and gender in Table 2.

16) For each group, number of participants (denominator) included in each analysis and whether the analysis was by original assigned groups

16-i) Report multiple "denominators" and provide definitions

Report multiple "denominators" and provide definitions: Report N's (and effect sizes) "across a range of study participation [and use] thresholds" [1], e.g., N exposed, N consented, N used more than x times, N used more than y weeks, N participants "used" the intervention/comparator at specific pre-defined time points of interest (in absolute and relative numbers per group). Always clearly define "use" of the intervention.

subitem not at all important

- 1 ○
- 2 ○
- 3 ◉
- 4 ○
- 5 ○

essential

Clear selection

Does your paper address subitem 16-i? *

Copy and paste relevant sections from the manuscript (include quotes in quotation marks "like this" to indicate direct quotes from your manuscript), or elaborate on this item by providing additional information not in the ms, or briefly explain why the item is not applicable/relevant for your study

We describe the available outcomes to be analyzed in the CONSORT flow diagram.

16-ii) Primary analysis should be intent-to-treat

Primary analysis should be intent-to-treat, secondary analyses could include comparing only “users”, with the appropriate caveats that this is no longer a randomized sample (see 18-i).

subitem not at all important

1 ○

2 ○

3 ◉

4 ○

5 ○

essential

Clear selection

Does your paper address subitem 16-ii?

Copy and paste relevant sections from the manuscript (include quotes in quotation marks "like this" to indicate direct quotes from your manuscript), or elaborate on this item by providing additional information not in the ms, or briefly explain why the item is not applicable/relevant for your study

"To assess 12-month intervention changes, we conducted complete-case analyses, excluding participants who did not complete the relevant 12-month assessment. Collapsing across all participants, we examined pre-post changes in HbA1c (our primary outcome), percent weight, PHQ-8, as well as the number of classes participants viewed, self-reported dietary adherence, and intervention satisfaction, using between subjects t-tests using SPSS 28.0 (IBM Corp., Armonk, N.Y., USA). We explored outcomes using intent-to-treat analyses (n=112) with linear mixed regression models using SPSS 28.0. This approach makes use of all available data at all timepoints, with the outcomes as dependent variables, time (pre, post), and all three strategies in a full-factorial design. We explored overall changes across strategies using within subjects t-tests using SPSS. "

17a) For each primary and secondary outcome, results for each group, and the estimated effect size and its precision (such as 95% confidence interval)

Does your paper address CONSORT subitem 17a? *

Copy and paste relevant sections from the manuscript (include quotes in quotation marks "like this" to indicate direct quotes from your manuscript), or elaborate on this item by providing additional information not in the ms, or briefly explain why the item is not applicable/relevant for your study

Yes, we report 95% confidence intervals.

17a-i) Presentation of process outcomes such as metrics of use and intensity of use

In addition to primary/secondary (clinical) outcomes, the presentation of process outcomes such as metrics of use and intensity of use (dose, exposure) and their operational definitions is critical. This does not only refer to metrics of attrition (13-b) (often a binary variable), but also to more continuous exposure metrics such as “average session length”. These must be accompanied by a technical description how a metric like a “session” is defined (e.g., timeout after idle time) [1] (report under item 6a).

subitem not at all important

1 ○

2 ○

3 ◉

4 ○

5 ○

essential

Clear selection

Does your paper address subitem 17a-i?

Copy and paste relevant sections from the manuscript (include quotes in quotation marks "like this" to indicate direct quotes from your manuscript), or elaborate on this item by providing additional information not in the ms, or briefly explain why the item is not applicable/relevant for your study

"Once participants were assigned to the different intervention strategies, we e-mailed all participants links to the core online VLC intervention materials throughout the 12-month intervention, weekly for the first 4 months and then every 2 weeks for the remaining 8 months, for a total of 32 emails. Each of the 32 sets of materials focused on a different topic related to following a VLC diet..."

17b) For binary outcomes, presentation of both absolute and relative effect sizes is recommended

Does your paper address CONSORT subitem 17b? *

Copy and paste relevant sections from the manuscript (include quotes in quotation marks "like this" to indicate direct quotes from your manuscript), or elaborate on this item by providing additional information not in the ms, or briefly explain why the item is not applicable/relevant for your study

We report this information in the tables.

18) Results of any other analyses performed, including subgroup analyses and adjusted analyses, distinguishing pre-specified from exploratory

Does your paper address CONSORT subitem 18? *

Copy and paste relevant sections from the manuscript (include quotes in quotation marks "like this" to indicate direct quotes from your manuscript), or elaborate on this item by providing additional information not in the ms, or briefly explain why the item is not applicable/relevant for your study

We describe other outcomes, such as qualitative descriptions.

18-i) Subgroup analysis of comparing only users

A subgroup analysis of comparing only users is not uncommon in ehealth trials, but if done, it must be stressed that this is a self-selected sample and no longer an unbiased sample from a randomized trial (see 16-iii).

subitem not at all important

1 ○

2 ○

3 ●

4 ○

5 ○

essential

Clear selection

Does your paper address subitem 18-i?

Copy and paste relevant sections from the manuscript (include quotes in quotation marks "like this" to indicate direct quotes from your manuscript), or elaborate on this item by providing additional information not in the ms, or briefly explain why the item is not applicable/relevant for your study

We did not report on a subgroup analysis.

19) All important harms or unintended effects in each group
(for specific guidance see CONSORT for harms)

Does your paper address CONSORT subitem 19? *

Copy and paste relevant sections from the manuscript (include quotes in quotation marks "like this" to indicate direct quotes from your manuscript), or elaborate on this item by providing additional information not in the ms, or briefly explain why the item is not applicable/relevant for your study

"No serious adverse events were reported during the trial." "We examined qualitative comments about other changes and impacts...."

19-i) Include privacy breaches, technical problems

Include privacy breaches, technical problems. This does not only include physical "harm" to participants, but also incidents such as perceived or real privacy breaches [1], technical problems, and other unexpected/unintended incidents. "Unintended effects" also includes unintended positive effects [2].

subitem not at all important

1 ○

2 ○

3 ◉

4 ○

5 ○

essential

Clear selection

Does your paper address subitem 19-i?

Copy and paste relevant sections from the manuscript (include quotes in quotation marks "like this" to indicate direct quotes from your manuscript), or elaborate on this item by providing additional information not in the ms, or briefly explain why the item is not applicable/relevant for your study

We did not describe these as they did not occur.

19-ii) Include qualitative feedback from participants or observations from staff/researchers

Include qualitative feedback from participants or observations from staff/researchers, if available, on strengths and shortcomings of the application, especially if they point to unintended/unexpected effects or uses. This includes (if available) reasons for why people did or did not use the application as intended by the developers.

subitem not at all important

1 ○

2 ○

3 ◉

4 ○

5 ○

essential

Clear selection

Does your paper address subitem 19-ii?

Copy and paste relevant sections from the manuscript (include quotes in quotation marks "like this" to indicate direct quotes from your manuscript), or elaborate on this item by providing additional information not in the ms, or briefly explain why the item is not applicable/relevant for your study

"We explored participants' perceptions of the three supplemental strategies using open-ended questions on the self-report survey...

Participants also commented on the mindful eating and positive affect skills...

We examined qualitative comments about other changes and impacts..."

DISCUSSION

22) Interpretation consistent with results, balancing benefits and harms, and considering other relevant evidence

NPT: In addition, take into account the choice of the comparator, lack of or partial blinding, and unequal expertise of care providers or centers in each group

22-i) Restate study questions and summarize the answers suggested by the data, starting with primary outcomes and process outcomes (use)

Restate study questions and summarize the answers suggested by the data, starting with primary outcomes and process outcomes (use).

subitem not at all important

1 ○

2 ○

3 ◉

4 ○

5 ○

essential

Clear selection

Does your paper address subitem 22-i? *

Copy and paste relevant sections from the manuscript (include quotes in quotation marks "like this" to indicate direct quotes from your manuscript), or elaborate on this item by providing additional information not in the ms, or briefly explain why the item is not applicable/relevant for your study

"This study addressed improving outcomes in an online VLC intervention for adults with type 2 diabetes by using the MOST framework to examine which of three potentially helpful supplemental behavioral strategies significantly contributed to reduced HbA1c and other outcomes. Although HbA1c decreased for both levels of all components tested, no differences were statistically significant. "

22-ii) Highlight unanswered new questions, suggest future research

Highlight unanswered new questions, suggest future research.

subitem not at all important

1 ○

2 ○

3 ◉

4 ○

5 ○

essential

Clear selection

Does your paper address subitem 22-ii?

Copy and paste relevant sections from the manuscript (include quotes in quotation marks "like this" to indicate direct quotes from your manuscript), or elaborate on this item by providing additional information not in the ms, or briefly explain why the item is not applicable/relevant for your study

"The addition of monthly (not daily) dietary self-monitoring, mindful eating, and positive affect skills training did not show definitive benefit but are worth testing further."

20) Trial limitations, addressing sources of potential bias, imprecision, and, if relevant, multiplicity of analyses

20-i) Typical limitations in ehealth trials

Typical limitations in ehealth trials: Participants in ehealth trials are rarely blinded. Ehealth trials often look at a multiplicity of outcomes, increasing risk for a Type I error. Discuss biases due to non-use of the intervention/usability issues, biases through informed consent procedures, unexpected events.

subitem not at all important

1 ○

2 ○

3 ◉

4 ○

5 ○

essential

Clear selection

Does your paper address subitem 20-i? *

Copy and paste relevant sections from the manuscript (include quotes in quotation marks "like this" to indicate direct quotes from your manuscript), or elaborate on this item by providing additional information not in the ms, or briefly explain why the item is not applicable/relevant for your study

"A limitation of the trial was that 80% of the sample were women... Moreover, we could only test a finite set of supplementary strategies, so we cannot generalize our conclusions beyond the strategies tested. "

21) Generalisability (external validity, applicability) of the trial findings

NPT: External validity of the trial findings according to the intervention, comparators, patients, and care providers or centers involved in the trial

21-i) Generalizability to other populations

Generalizability to other populations: In particular, discuss generalizability to a general Internet population, outside of a RCT setting, and general patient population, including applicability of the study results for other organizations

subitem not at all important

1 ○

2 ○

3 ◉

4 ○

5 ○

essential

Clear selection

Does your paper address subitem 21-i?

Copy and paste relevant sections from the manuscript (include quotes in quotation marks "like this" to indicate direct quotes from your manuscript), or elaborate on this item by providing additional information not in the ms, or briefly explain why the item is not applicable/relevant for your study

""A limitation of the trial was that 80% of the sample were women..."

21-ii) Discuss if there were elements in the RCT that would be different in a routine application setting

Discuss if there were elements in the RCT that would be different in a routine application setting (e.g., prompts/reminders, more human involvement, training sessions or other co-interventions) and what impact the omission of these elements could have on use, adoption, or outcomes if the intervention is applied outside of a RCT setting.

subitem not at all important

1 ○

2 ○

3 ◉

4 ○

5 ○

essential

Clear selection

Does your paper address subitem 21-ii?

Copy and paste relevant sections from the manuscript (include quotes in quotation marks "like this" to indicate direct quotes from your manuscript), or elaborate on this item by providing additional information not in the ms, or briefly explain why the item is not applicable/relevant for your study

We did not describe this information as it is not directly relevant.

OTHER INFORMATION

23) Registration number and name of trial registry

Does your paper address CONSORT subitem 23? *

Copy and paste relevant sections from the manuscript (include quotes in quotation marks "like this" to indicate direct quotes from your manuscript), or elaborate on this item by providing additional information not in the ms, or briefly explain why the item is not applicable/relevant for your study

"ClinicalTrials.gov NCT03037528"

24) Where the full trial protocol can be accessed, if available

Does your paper address CONSORT subitem 24? *

Cite a Multimedia Appendix, other reference, or copy and paste relevant sections from the manuscript (include quotes in quotation marks "like this" to indicate direct quotes from your manuscript), or elaborate on this item by providing additional information not in the ms, or briefly explain why the item is not applicable/relevant for your study

The full trial protocol is not available.

25) Sources of funding and other support (such as supply of drugs), role of funders

Does your paper address CONSORT subitem 25? *

Copy and paste relevant sections from the manuscript (include quotes in quotation marks "like this" to indicate direct quotes from your manuscript), or elaborate on this item by providing additional information not in the ms, or briefly explain why the item is not applicable/relevant for your study

"LS and this research were supported by a K01 award from the National Institutes of Health (NIDDK, NIH), DK107456, from the National Institute of Diabetes and Digestive and Kidney Diseases...."

X27) Conflicts of Interest (not a CONSORT item)

X27-i) State the relation of the study team towards the system being evaluated

In addition to the usual declaration of interests (financial or otherwise), also state the relation of the study team towards the system being evaluated, i.e., state if the authors/evaluators are distinct from or identical with the developers/sponsors of the intervention.

subitem not at all important

1 ○

2 ○

3 ◉

4 ○

5 ○

essential

Clear selection

Does your paper address subitem X27-i?

Copy and paste relevant sections from the manuscript (include quotes in quotation marks "like this" to indicate direct quotes from your manuscript), or elaborate on this item by providing additional information not in the ms, or briefly explain why the item is not applicable/relevant for your study

"LS's partner, HB, is an inventor of software used in this study, which purchased a software services agreement for its use."

About the CONSORT EHEALTH checklist

As a result of using this checklist, did you make changes in your manuscript? *

- ○ yes, major changes
- ◉ yes, minor changes
- ○ no

What were the most important changes you made as a result of using this checklist?

I'm not sure which were the most important.

How much time did you spend on going through the checklist INCLUDING making changes in your manuscript *

I spent two hours making these changes.

As a result of using this checklist, do you think your manuscript has improved? *

- ◉ yes
- ○ no
- ○ Other:

Would you like to become involved in the CONSORT EHEALTH group?

This would involve for example becoming involved in participating in a workshop and writing an "Explanation and Elaboration" document

- ○ yes
- ● no
- ○ Other:

Clear selection

Any other comments or questions on CONSORT EHEALTH

Requiring all responses to be at least 25 characters is unnecessary.

STOP - Save this form as PDF before you click submit

To generate a record that you filled in this form, we recommend to generate a PDF of this page (on a Mac, simply select "print" and then select "print as PDF") before you submit it.

When you submit your (revised) paper to JMIR, please upload the PDF as supplementary file.

Don't worry if some text in the textboxes is cut off, as we still have the complete information in our database. Thank you!

Final step: Click submit !

Click submit so we have your answers in our database!

Submit

Clear form

Never submit passwords through Google Forms.

This form was created outside of your domain. Report Abuse - Terms of Service - Privacy Policy

Google Forms
